# Toward clinically relevant automated corneal biomanufacturing with human-derived FBS alternatives

**DOI:** 10.1038/s41598-026-58401-5

**Published:** 2026-06-17

**Authors:** Alexandre Taoum, Julia S. Oster, Ole Thaden, Andrea Frank, Meng Wang, Mario Wisbar, Matthias Fuest, Friederike Dehli, Daniela F. Duarte Campos

**Affiliations:** 1 Bioprinting & Tissue Engineering Group, Center for Molecular Biology of Heidelberg University, Heidelberg, Germany; 2 Department of Ophthalmology, RWTH Aachen University, Aachen, Germany

**Keywords:** FBS alternatives, Human platelet lysate, Human serum, Keratocyte differentiation, Corneal tissue engineering, Xeno-reduced culture systems, Biological techniques, Biotechnology, Cell biology, Medical research, Stem cells

## Abstract

The replacement of animal-derived components from cell culture protocols is essential for the development of human-compatible and clinically translatable systems. Fetal bovine serum (FBS) is still widely used for cell expansion, although its xenogeneic origin and batch variability limit regulatory compliance. In this study, human platelet lysate (hPL) and human serum (HS) were assessed as low-serum (2%) alternatives to FBS during the subculture phase of bone marrow-derived mesenchymal stromal cells (BM-MSC) prior to keratocyte differentiation for corneal tissue engineering. BM-MSC were gradually adapted over four days to 2% FBS, hPL, or HS, maintained for seven days, and then transferred to serum-free keratocyte differentiation medium for 14 days in either two-dimensional (2D) or three-dimensional (3D) cultures in 30 wt% riboflavin-arginine-triggered gelatin methacryloyl (RA-GelMA) hydrogels crosslinked under visible blue light. To demonstrate the applicability of this approach in corneal tissue engineering, differentiation was evaluated by immunofluorescence and quantitative PCR in 2D cultures, and by immunofluorescence in 3D cultures. Both hPL and HS maintained metabolic activity, supported keratocyte-associated marker expression, and suppressed α-SMA, performing comparably to FBS, thereby supporting their use as clinically compliant, human-derived alternatives for xeno-reduced corneal biomanufacturing.

**Supplementary Information:**

The online version contains supplementary material available at 10.1038/s41598-026-58401-5.

## Introduction

Corneal diseases are a major cause of visual impairment worldwide^1^. Keratoplasty, also known as corneal grafting, remains the gold standard procedure for treating corneal blindness^2^. However, donor shortage, graft rejection, and post-operative complications limit its availability and long-term supply^3–5^, with a growing demand for available corneas^6^.

Tissue-engineered stromal equivalents have therefore been investigated to restore corneal transparency and function by replicating the native extracellular matrix (ECM) architecture and cellular phenotype^7–9^. While advanced models increasingly aim to reproduce multiple corneal layers, including epithelial and stromal compartments, the stromal layer remains a major target for tissue engineering^10^. The corneal epithelium is a self-renewing tissue maintained by stem cells localized in the peripheral limbus^11^ and can rapidly regenerate following injury^12^. In contrast, stromal injuries frequently result in fibrosis and permanent loss of transparency^13^.

In the tissue engineering field, and more generally the cell culture area, fetal bovine serum (FBS) remains the most widely used supplement for cell expansion. FBS constitutes a complex and partially undefined mixture of bioactive components^14^, including growth factors (e.g., TGF-β, FGF, PDGF), hormones, carrier proteins, lipids, and extracellular vesicles^15–17^, which collectively regulate cell behavior through receptor-mediated signaling pathways, endocytosis, and modulation of extracellular matrix interactions, thereby influencing proliferation, survival, and lineage commitment^18^.

However, its xenogeneic origin, undefined composition, and batch-to-batch variability are raising significant safety and regulatory concerns, including the risk of zoonotic disease transmission and immunogenicity^19–21^. For these reasons, the replacement of FBS with human-derived or chemically defined supplements is considered essential for the development of animal-free systems, following the Good Manufacturing Practice (GMP)^22,23^, which requires strict control of raw materials, traceability, and reproducibility, thereby discouraging animal-derived incompatible with clinical-grade manufacturing standards^24^.

Human platelet lysate (hPL) is obtained from pooled platelet concentrates subjected to freeze–thaw cycles to release platelet-derived growth factors and cytokines^25^. In contrast, Human Serum (HS) is the liquid fraction obtained after coagulation of whole blood collected without anticoagulants, followed by centrifugation to remove the clot and cellular components. During coagulation, fibrinogen and other clotting factors are consumed, resulting in a protein-rich supplement^26,27^. While both hPL and HS support cell growth, their composition differs. hPL is rich in platelet-derived growth factors such as PDGF, TGF-β, and VEGF, which strongly stimulate cell proliferation^22,28^. In contrast, HS contains higher levels of serum proteins, immunoglobulins, and complement factors, which are involved in cell adhesion, transport, and immune-related functions^29^. Their use as culture supplements offers two main advantages: elimination of xenogeneic components and the potential for autologous or allogeneic use, thus reducing immunological and infectious risks^30,31^.

Bone marrow-derived mesenchymal stromal cells (BM-MSC) are widely used in regenerative medicine due to their accessibility, immunomodulatory properties, and multilineage differentiation potential^32,33^. Recently, BM-MSC have emerged as a relevant cell source for corneal stromal tissue engineering due to their ability to differentiate toward keratocyte lineages^34^. In our previous study, we showed that 3D culture systems provide a more physiologically relevant environment than 2D culture for differentiation of BM-MSC towards Corneal Stromal Keratocytes (CSK) lineages^35^.

Gelatin methacryloyl (GelMA) hydrogels are suited to these applications due to their biocompatibility and tunable mechanical properties^36^. Recently, a novel biobased photocrosslinking system for GelMA using riboflavin as a photosensitizer and arginine as co-initiator (RA-GelMA), has been developed for the 3D encapsulation and culture of primary human corneal keratocytes^37^. However, the differentiation of BM-MSC in RA-GelMA has not yet been assessed.

**Fig. 1:**
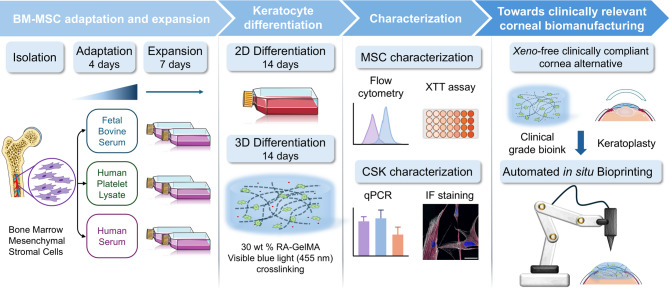
Schematic overview of the experimental workflow and future translational applications. Human BM-MSC were isolated and gradually adapted over 4 days to culture media supplemented with fetal bovine serum (FBS), human platelet lysate (hPL), or human serum (HS), followed by 7 days of expansion in their respective conditions. Cells were then differentiated toward a corneal stromal keratocyte-like phenotype in parallel using either 2D culture or 3D RA-GelMA hydrogels under serum-free keratocyte differentiation conditions for 14 days. Cell phenotype and differentiation were assessed using flow cytometry, XTT assay, qPCR, and immunofluorescence staining. The final panel illustrates the potential future application of these xeno-reduced culture systems for clinically relevant corneal biomanufacturing and automated in situ bioprinting approaches. Image adapted from Servier Medical Art (https://smart.servier.com/), licensed under CC BY 4.0 (https://creativecommons.org/licenses/by/4.0/).

In this study, for the first time, hPL and HS were evaluated as low serum (2%) replacements for FBS during BM-MSC expansion prior to CSK differentiation (Fig. 1). Differentiation was induced under serum free conditions in both 2D cultures and 3D RA-GelMA hydrogels. Outcomes were assessed by quantitative PCR, immunofluorescence, and morphological analysis in 2D cultures, while differentiation within 3D RA-GelMA constructs was assessed by immunofluorescence and morphological analysis. Corneal stromal markers (Lumican, Keratocan, ALDH3A1, and ALDH1A1) and the myofibroblast marker α-SMA were evaluated^38,39^. These investigations provide new insight into how human-derived expansion conditions impact the BM-MSC differentiation towards MSC-derived keratocytes (CSK-MSC), contributing to the development of animal-free, clinically relevant workflows for corneal tissue engineering.

## Materials and methods

### Cell culture

Human BM-MSC from three Caucasian donors (female, 62 years; male, 63 years; female, 72 years) were purchased from PromoCell (C-12974, PromoCell, Germany) and initially maintained in the isolation medium provided by the supplier, PromoCell Mesenchymal Stromal Cell Growth Medium 2 (MSCcom, C-28009, PromoCell, Germany) supplemented with 1% penicillin–streptomycin (Pen/Strep) (P4333, Sigma Aldrich, Germany), to allow cell recovery and stabilization after thawing. Cells from the three donors were pooled and seeded on collagen-coated plates at 5,000 cells cm^–2^.

Three experimental conditions were established in parallel using Mesenpan Basal medium (P08-50400B, PAN-Biotech, Germany) supplemented with 1% Mesenpan Growth Supplement (P08-5040S1, PAN-Biotech, Germany), 200 µM L-glutamine (G7513, Sigma Aldrich, Germany), 1% Pen/Strep, and one of the following serum supplements: FBS Supreme (P30-3031, PAN-Biotech, Germany), hPL ELAREM™ Perform-FD PLUS, Research Grade (PE30611, PL BioScience, Germany), or HS (P30-2401, PAN-Biotech, Germany). To minimise adaptation stress^40^, cells were transitioned from MSCcom to their respective Mesenpan formulation over three days, using sequential 75:25, 50:50, and 25:75 mixtures of the current to target medium, followed by 100% target medium from day 4. Cultures were maintained in their respective conditions for a further 7 days, with medium changes every 2 days, prior to keratocyte differentiation. During this period, cells were passaged when required to prevent overconfluence.

At the end of the adaptation phase, cells were seeded to reach approximately 70% confluency, and keratocyte differentiation was induced by replacing the culture medium with keratocyte differentiation (KD) medium consisting of DMEM/F12 (P04-41150, Pan Biotech, Germany), 1% MEM Vitamin Solution (M6895, Sigma-Aldrich, Germany), 1% MEM Non-Essential Amino Acids (M7145, Sigma-Aldrich, Germany), 1% Insulin-Transferrin-Selen (41400045, Gibco, Germany) 1 mM L-Ascorbate 2-Phosphate (LA2P, A8960, Merck, Germany), 20 ng/ml human recombinant Fibroblast Growth Factor-2 (FGF-2, CB-1102021, Pan Biotech, Germany), 0.1 ng/ml human recombinant Transforming Growth Factor-β3 (78131, Stem Cell Technologies, Germany) and 1% Pen/Strep. KD medium was changed every 2 days. MSC-derived keratocytes (CSK-MSC) were collected day 14 of differentiation by detaching with 1X accutase solution (A6964, Sigma Aldrich, Germany).

Human Corneal Keratocytes (HCK) were purchased from Innoprot (P10872, Innoprot, Spain) and subcultured on collagen coated plates in CSK basal medium made of Dulbecco’s Minimum Essential Medium DMEM/F12 supplemented with 0.5% Fetal Bovine Serum (FBS) (P30-3031, Pan Biotech, Germany), 1% MEM Vitamin Solution, 1% MEM Non-Essential Amino Acids), 1 mM LA2P, 10 µM ROCK-inhibitor (688002-M, Millipore, Germany), 10 ng/ml Recombinant Human Insulin-like Growth Factor-1 (CB-1104113, Pan Biotech, Germany), and 1% Pen/Strep.

All cell cultures were carried on T75 cell culture flasks (83.3911.002, Sarstedt, Germany) coated with type I collagen from calf skin (L7213, Sigma–Aldrich, Germany). A coating solution of 80 µg mL^− 1^, was prepared from the 4 mg mL^− 1^, collagen stock diluted in sterile 1X phosphate-buffered saline (PBS, 14190250, Gibco, Germany) and applied at a final surface density of 10 µg cm^− 2^. Flasks were incubated with the coating solution for 1 h at 37 °C before cell culture experiments.

### Hydrogel preparation

BM-MSC were encapsulated in a photocrosslinkable RA-GelMA hydrogel prepared according to the protocol of Dehli et al., 2025. Briefly, 25.75 g gelatin type B (G9382, Sigma Aldrich, Germany) was dissolved in 250 mL deionized water at 40 °C under continuous stirring, followed by the addition of 13 mL methacrylic anhydride (276685, Sigma Aldrich, Germany)^41^. The pH was adjusted to 7.3 using an automatic titrator (T50, Mettler Toledo, Germany) with 4 M NaOH (1310-73-2, Sigma Aldrich, Germany). After 5 h of reaction maintained under continuous stirring, the mixture was filtered and stored at 8 °C for 2 days. Purification was carried out by dialysis using a 12–14 kDa molecular weight cutoff membrane (2720.1, Carl Roth, Germany) against deionized water for 5 days, with two water changes per day. The degree of methacryloylation was quantified by H-NMR spectroscopy (300 MHz) in deuterium oxide D₂O (6672.7, Carl Roth, Germany) with trimethylsilylpropanoic acid (TSP) as the internal standard. The GelMA used in this study had a degree of methacryloylation of 0.52 mmol g^− 1^.

Prior to encapsulation, riboflavin (CDS025203, Sigma Aldrich, Germany) was first dissolved in Mesenpan medium at a final concentration of 190 µmol L^− 1^, followed by the addition of L-Arginine Monohydrochloride (A5131, Sigma Aldrich, Germany) at a final concentration of 80 mmol L^− 1^. GelMA was then dissolved in this solution at 37 °C to obtain a final concentration of 30 wt% and mixed by vortexing for 5 min until complete dissolution. BM-MSC were then suspended in the bioink at a concentration of 2 × 10^6^ cells mL^− 1^. The RA-GelMA bioink was pipetted in a cylindrical mold with a height of 2 mm and a diameter of 4 mm. Crosslinking was carried out for 10 min with blue light (455 nm LED, Thorlabs, Germany) at an intensity of 4.3 mW cm^− 2^. After crosslinking, the hydrogels were individually transferred into 24-well plates (83.3922.005, Sarstedt, Germany) and maintained fully immersed in1 mL of their respective cell culture medium and incubated for 1 h. The medium was then refreshed and replaced every two days throughout the culture period.

Differentiation of the samples was conducted following the protocol of Taoum et al. 2025^35^. 7 days after crosslinking and culture in their respective media, samples were immersed during 14 days in KD medium, with media changes every 2 days. All experimental conditions were performed in three technical triplicates (*n* = 3).

### Cell metabolic activity assay

Cell metabolic activity was assessed using the CyQUANT™ XTT Cell Viability Assay (X12223, Thermo Fisher Scientific, USA). BM-MSC cultured in Mesenpan medium supplemented with 2% FBS, hPL, or HS were harvested and seeded at 2,000 cells per well in collagen-coated 96-well plates (83.3923, Sarstedt, Germany). For each condition, three wells containing only medium served as blanks. Metabolic activity was assessed at days 1, 4, and 7, corresponding to the number of days in culture with 100% Mesenpan medium after the gradual adaptation phase to the new FBS alternative. All experimental conditions, including blanks were performed in three technical triplicates (*n* = 3).

The XTT reagent was prepared by mixing 6 parts XTT solution with 1part electron coupling reagent following the manufacturer’s protocol, and 70 µL of the mixture was added per well. Plates were incubated for 2 h at 37 °C and 5% CO_2_, and absorbance was recorded at 450 nm and 660 nm using a Spark Cyto plate reader. Specific absorbance was calculated as:

$$A_{{{\mathrm{specific}}}} = \left( {A_{{450}} - A_{{660}} } \right)_{{{\mathrm{sample}}}} - \left( {A_{{450}} - A_{{660}} } \right)_{{{\mathrm{blank}}}}$$

### Flow cytometry

Following gradual adaptation, pooled BM-MSC were cultured for 7 days in Mesenpan supplemented with 2% FBS, hPL, or HS. Cells maintained in MSCcom medium served as control. For each condition, one million cells were harvested, washed in PBS containing 2% of the respective culture serum (FACS buffer), and stained with the MSC Characterization Antibody Panel (100–0354, Stemcell Technologies, Germany) for CD73, CD90, CD105, and the negative marker CD45, each conjugated to a distinct fluorophore. Staining was performed for 30 min at 4 °C, followed by washing and resuspension in FACS buffer. Unstained and single-stained controls were prepared using MSCcom cultured cells, and compensation for CD45 was performed using compensation beads (01-3333-41, Thermo Fisher, Germany). Samples were analysed on a BD FACSymphony™ A1 flow cytometer, and data were processed with BD FACSDiva software (Version 9.0.2, BD Biosciences, Germany). All experimental conditions were performed in three technical triplicates (*n* = 3).

### Immunofluorescence staining

CSK markers were assessed by immunofluorescence staining in 2D and 3D cultures. For 2D cultures, BM-MSC were seeded on collagen-coated µ-Plate 96 Well Round ibiTreat plates (89606, Ibidi GmbH, Germany) at 5,000 cells cm^−2^ (pre-differentiation) or 10,000 cells cm^−2^ (post-differentiation). For 3D cultures, RA-GelMA hydrogels containing BM-MSC were used after 7 days in respective MSC medium, and CSK-MSC were used directly after 14 days of differentiation. Samples were washed twice with PBS containing 1 mM MgCl₂, fixed with 4% paraformaldehyde (47347.9 M, VWR, Germany) in 1X PBS (10 min for 2D, 20 min for 3D), and washed three times with PBS + 1 mM MgCl_2_. Permeabilization was performed with PBS containing 0.2% Triton X-100 (1086031000, VWR, Germany), followed by blocking in PBS + 1 mM MgCl_2_ supplemented with 5% goat serum (P30-1001, PAN Biotech, Germany), 5% donkey serum (P30‐0101, PAN Biotech, Germany), and 0.1% Triton X-100 for 60 min (2D) or 120 min (3D).

Primary antibodies (Table 1) were diluted in blocking buffer and incubation was carried out for 2 h (2D) or 4 h (3D) at room temperature, followed by three washes in PBS containing 0.2% Tween-20. Secondary antibodies, Hoechst, and Phalloidin (Table 1) were applied for 1 h (2D) or 4 h (3D) under the same respective conditions. All incubation times were doubled in 3D compared to 2D to allow diffusion of the solution within the constructs. After final washes, 2D samples were kept in PBS at 4 °C until imaging, and 3D gels were placed in Ibidi µ-Dishes (81158, Ibidi GmbH, Germany). All experimental conditions in 2D and 3D, including backgrounds, were performed in three technical triplicates (*n* = 3).

All samples were imaged using a Zeiss LSM780 confocal microscope with ZEN 2011 SP2 software. Images were processed in Imaris (Version 10.2.0, Oxford Instruments) using deconvolution and background subtraction. Background controls without primary antibody were conducted for all staining conditions to assess non-specific secondary antibody signal and to adjust imaging parameters accordingly. Immunofluorescence staining was used as a qualitative assessment of CSK marker expression.

**Table 1:** Antibody list and dilution factors.

Primary antibody		
Target antigen	Host	Dilution factor
α-SMA (MA5-11547, Thermo Fisher, Germany)	Mouse	1:500 (0.8 µg ml^− 1^)
ALDH1A1 (60171-1-Ig, Proteintech, Germany)	Mouse	1:500 (2 µg ml^− 1^)
ALDH3A1 (68036-1-Ig, Proteintech, Germany)	Mouse	1:400 (2.5 µg ml^− 1^)
Keratocan (bs-11054R, Thermo Fisher, Germany)	Rabbit	1:200 (5 µg ml^− 1^)
Lumican (bs-5890R, Thermo Fisher, Germany)	Rabbit	1:200 (5 µg ml^− 1^)
Collagen I (14695-1-AP, Proteintech, Germany)	Rabbit	1:400 (1.5 µg ml^− 1^)

Secondary antibody		
Fluorophore	Reactivity	Dilution factor
Alexa Fluor 594 (ab150080, abcam, Germany)	Rabbit	1:400 (2 µg ml^− 1^)
Alexa Fluor 647 (ab150115, abcam, Germany)	Mouse	1:500 (2 µg ml^− 1^)
Hoechst (62249, Fisher scientific, Germany)		1:500 (1 µg ml^− 1^)
Alexa Fluor 488 Phalloidin (A12379, Thermo Fischer, Germany)		1:500 (0.12 µg ml^− 1^)

### qPCR analysis

qPCR analyses were conducted in 2D cultures. Cells were washed twice with PBS and collected by centrifugation at 12,000 × g. The pellet was then snap-frozen in liquid nitrogen prior to RNA extraction. Total RNA was isolated using the NucleoSpin RNA Plus kit (740984.50, Macherey-Nagel, Germany) according to the manufacturer’s protocol, including the integrated genomic DNA removal step.

cDNA synthesis was performed using a synthesis kit (RevertAid First Strand cDNA Synthesis, K1622, Thermo Fisher, Germany). Gene expression was quantified by qRT-PCR reactions, performed on 10 µg cDNA using the Sso Advanced Universal SYBR Green Supermix (1725270 Bio-Rad, US) and the Bio-Rad CFX Opus 384 Thermocycler following the program described in Table 2. All experimental conditions were performed in three technical triplicates (*n* = 3).

**Table 2:** qPCR cycling program.

Cycle step	Temperature [°C]	Time [s]	Cycles
Polymerase activation and initial denaturation	95	30	1
Denaturation	95	10	40
Annealing and extension	62	15	40
Plate read	62	-	40
Melt curve	65–95 (0.5 increments)	5	1

Fold change was calculated by the $2^{{ - {{\Delta \Delta Ct}}}}$ method.

$$\Delta Ct = Ct\left( {Target} \right) - Ct\left( {Reference} \right)$$

$$\Delta \Delta Ct = ~\Delta Ct\left( {Sample} \right) - \Delta Ct\left( {Control} \right)$$

$$Fold~change = 2^{{ - \Delta \Delta Ct}}$$

Glyceraldehyde 3-phosphate dehydrogenase (GAPDH) was used as housekeeping gene and no-template controls (NTC) were included in all qPCR assays to confirm the absence of contamination and non-specific amplification. Primer efficiency was determined for all primer pairs using a five-point 1:2 serial dilution of pooled cDNA. Briefly, cDNA obtained from two reverse transcription reactions was pooled and serially diluted from 1:2 to 1:32 using nuclease-free water. qPCR reactions were performed in three technical triplicates (*n* = 3). Primer efficiency and corresponding R² values were calculated from the standard curves generated by plotting Ct values against the logarithm of the relative cDNA dilution factor.

The range of the fold change was then calculated based on the standard deviations (s) from the Ct values.

$$2^{{ - \left( {\Delta \Delta Ct \pm s} \right)}} ,\;~with\;~s = \sqrt {\left( {s_{{target}} } \right)^{2} - \left( {s_{{reference}} } \right)^{2} }$$

The level of expression of CSK markers before and after differentiation in 2D cultures was compared to the level of expression of these markers in BM-MSC cultivated in MSC medium. The level of expression was normalized to BM-MSC prior to differentiation in 2D cultures. Primer sequences, PCR efficiencies, and corresponding R² values are summarized in Table 3. Although minor variations in amplification efficiency were observed between primer pairs, all assays demonstrated sufficient performance for relative quantification using the 2^−ΔΔCt^ method.

**Table 3:** qPCR primers.

Target	Forward primer	Reverse primer	Efficiency	*R*²
α-SMA	GAGACCCTGTTCCAGCCATC	CGTGATCTCCTTCTGCATTCG	102.85%	0.99808
ALDH1A1	AAAGCCATAACAATCTCCTCTG	TACTCTCCCAGTTCTCTTCC	80.84%	0.98255
ALDH3A1	GACTACATCCTCTGTGACCC	CCTTCTGGCCCTCAATCAG	91.13%	0.99510
Keratocan	GAAAAAGGAGCCCTAAGCCA	CTCCAGATTGCTAAAGGTCCCT	108.88%	0.94649
Lumican	CAAGACAGTAAGGATTCAAACC	TACCACCAATCAATGCCAG	93.14%	0.99791
GAPDH	GTCAAGGCTGAGAACGGGAA	CTTTTGGCTCCCCCCTGCAAAT	94.73%	0.99660

### Statistical analysis

All results were presented as mean ± standard deviation (SD) with the corresponding sample size described in the respective figure captions. Comparisons between multiple experimental groups with two factors were conducted using an ordinary one-way ANOVA with Tukey’s post hoc test (Prism 10.1.2, GraphPad Software, Boston, USA). Statistical significance was labeled as *p < 0.05, **p < 0.01, ***p < 0.001, and **** *p* < 0.0001.

## Results

### Human-derived serum alternatives promote increased metabolic activity after cell seeding

**Fig. 2:**
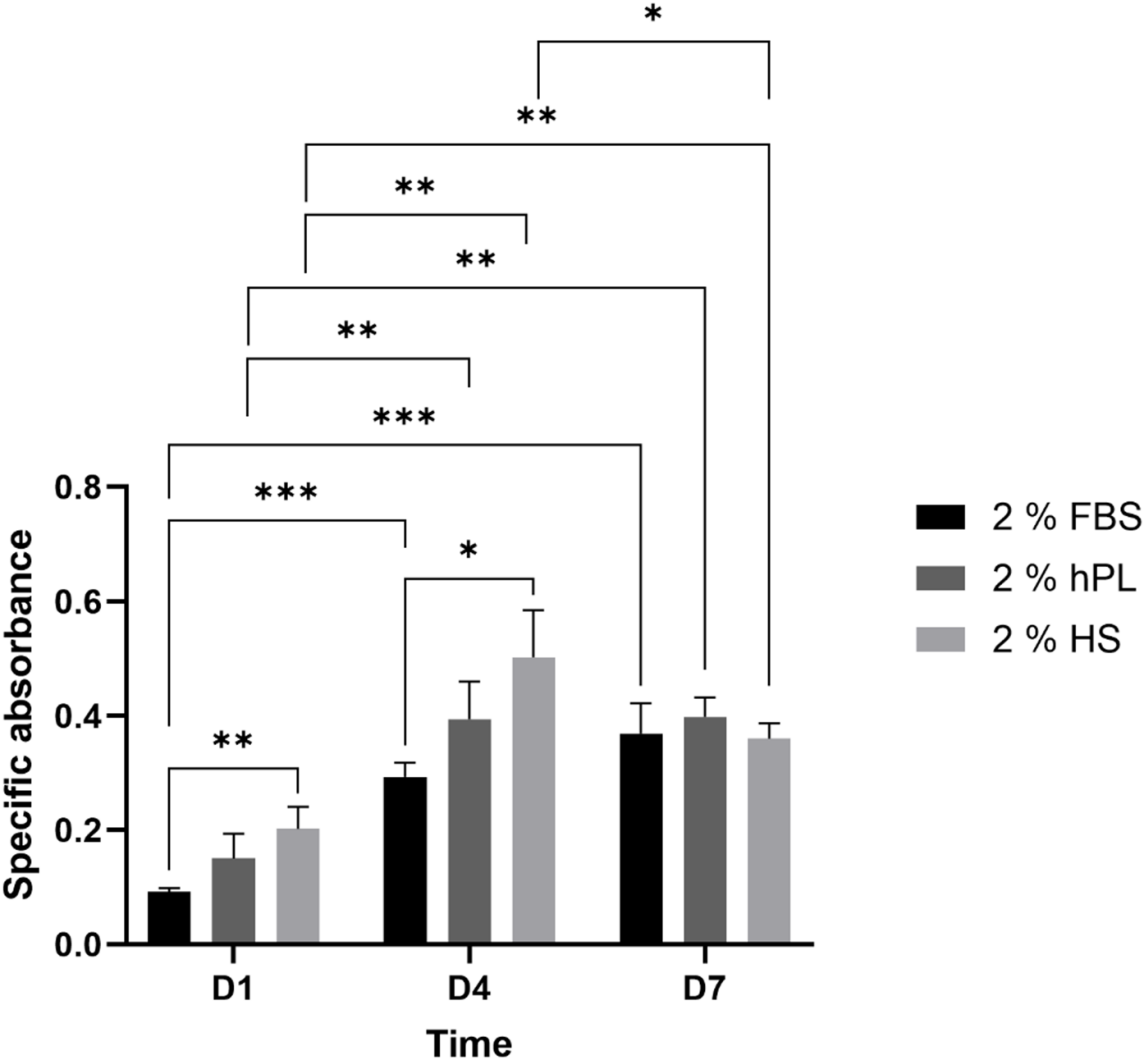
Metabolic activity of pooled BM-MSC cultivated in Mesenpan with 2% FBS, 2% hPL, or 2% HS at different timepoints. Timepoints are days after seeding cells with a density of 2,000 cells per well of a 96-well plate. Specific absorbance is measured 2 h after incubation with the XTT reagent. Data are presented as mean ± standard deviation of three technical replicates (*n* = 3). * p-value < 0.05, ** p-value < 0.01, *** p-value < 0.001, all other differences are not significant.

The XTT assay was conducted on days 1, 4, and 7 after cell seeding to evaluate the metabolic activity of BM-MSC cultured in Mesenpan medium supplemented with different serum alternatives (Fig. 2).

In all conditions, metabolic activity increased significantly from day 1 to day 4. Mean absorbance values increased from 0.0925 ± 0.0021 to 0.2929 ± 0.0236 in the FBS condition (*p* = 0.0002), from 0.1505 ± 0.0397 to 0.3944 ± 0.0626 in the hPL group (*p* = 0.0058), and from 0.2023 ± 0.0349 to 0.5021 ± 0.0792 in the HS condition (*p* = 0.0047).

Between days 4 and 7, only a slight additional increase was observed in the FBS condition (0.3692 ± 0.0487). Metabolic activity remained stable in the hPL condition (0.3982 ± 0.0280), whereas a significant decrease was measured in the HS condition (0.3604 ± 0.0184, *p* = 0.0480). Despite this decline, all conditions still showed an overall significant increase in metabolic activity from day 1 to day 7 (FBS *p* = 0.0009, hPL *p* = 0.0014, HS *p* = 0.0043).

At early time points (days 1 and 4), cells cultured with hPL and HS exhibited higher metabolic activity than those cultured with FBS. This difference was statistically significant for HS compared with FBS on day 1 (*p* = 0.0081) and day 4 (*p* = 0.0139). By day 7, no significant differences in metabolic activity were observed among the three conditions.

### Human-derived serum-supplemented culture media enable MSC to meet the minimum criteria of their characteristic surface markers

**Fig. 3:**
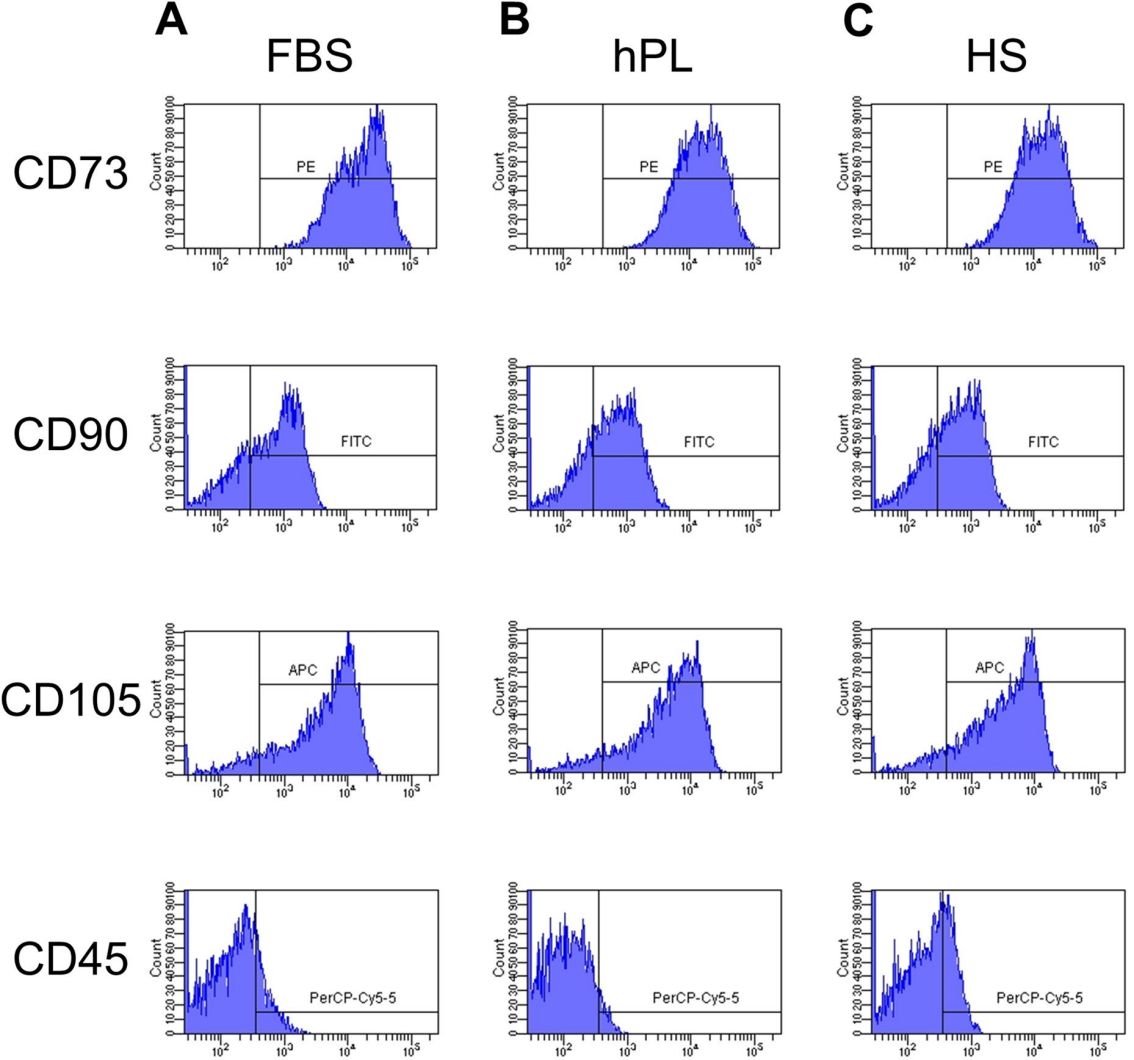
Flow cytometry analysis of BM-MSC surface marker expression after expansion in Mesenpan supplemented with different serum conditions. Histograms show the expression of the MSC markers CD73, CD90, and CD105, and the hematopoietic marker CD45. (A) BM-MSC cultured with 2% FBS. (B) BM-MSC cultured with 2% hPL. (C) BM-MSC cultured with 2% HS. Representative flow cytometry histograms from one technical replicate. All conditions were performed in three technical triplicates (*n* = 3).

BM-MSC supplemented with different serum conditions were characterized by flow cytometry to assess the expression of MSC surface markers (Fig. 3).

Cells cultured with 2% FBS showed clear expression of the MSC markers CD73, CD90, and CD105, while CD45 was not detected. CD90 expression appeared lower compared to CD73 and CD105. A similar expression pattern was observed for BM-MSC cultured with 2% hPL, with positive expression of CD73, CD90, and CD105 and no detectable CD45 signal.BM-MSC cultivated with 2% HS also expressed CD73 and CD105, while CD90 showed lower expression levels, consistent with the other serum conditions. CD45 remained negative in this condition.

Overall, these results confirm that BM-MSC expanded with FBS, hPL, or HS retain a typical MSC immunophenotype, characterized by the expression of CD73, CD90, and CD105 and the absence of CD45^42^.

### Serum alternatives support keratocyte marker expression following differentiation

To validate the differentiation of BM-MSC towards CSK-MSC, the expression of Collagen 1, of the CSK markers ALDH1A1, ALDH3A1, Lumican, and Keratocan, and the myofibroblast marker αSMA were examined by IF. Single signals for all immunofluorescence stainings in 2D are provided in the supplementary data (Figs. S1-S6).

**Fig. 4:**
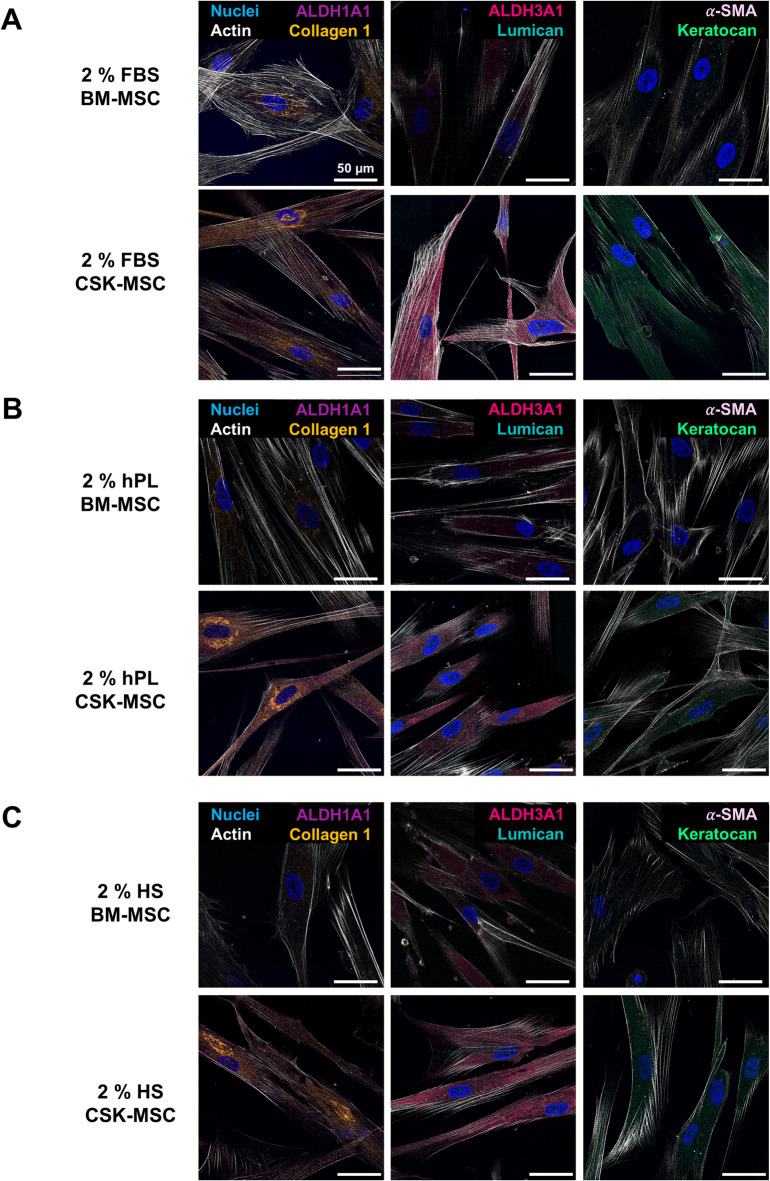
Immunostaining of 2D BM-MSC and CSK-MSC supplemented with different serum conditions before differentiation. Cells were stained for ALDH1A1 (light pink), Collagen I (orange), ALDH3A1 (dark pink), Lumican (turquoise), α-SMA (lilac), and Keratocan (green), with Hoechst nuclear staining (blue) and phalloidin actin staining (white). Scale bars represent 50 μm. Representative images from one technical replicate per antibody couple. All conditions were performed in three technical triplicates (*n* = 3). (A) Cells cultured with 2% FBS. Top: 2D BM-MSC before differentiation. Bottom: 2D CSK-MSC after differentiation. (B) Cells cultured with 2% hPL. Top: 2D BM-MSC before differentiation. Bottom: 2D CSK-MSC after differentiation. (C) Cells cultured with 2% HS. Top: 2D BM-MSC before differentiation. Bottom: 2D CSK-MSC after differentiation.

Before differentiation, BM-MSC cultured in Mesenpan medium supplemented with 2% FBS showed no detectable expression of ALDH1A1, Lumican, Keratocan, or α-SMA (Fig. 4A, top). A low basal signal for Collagen I was observed, and only a weak fluorescence signal for ALDH3A1 was detected.

After differentiation, cells previously expanded in FBS expressed the CSK-associated markers ALDH1A1, ALDH3A1, Lumican, and Keratocan (Fig. 4A, bottom). The Collagen I signal was detected after differentiation, and no expression of the myofibroblast marker α-SMA was detected.

A similar pattern was observed for BM-MSC expanded in Mesenpan medium with 2% hPL. Before differentiation, no expression of ALDH1A1, Lumican, Keratocan, or α-SMA was detected (Fig. 4B, top), while Collagen I and ALDH3A1 were detected. Following differentiation, cells expressed ALDH1A1, ALDH3A1, Lumican, and Keratocan (Fig. 4B, bottom). Collagen I staining was detected after differentiation, and α-SMA remained undetectable.

Before differentiation, BM-MSC cultured with 2% HS showed a marker expression profile comparable to that observed in the FBS and hPL conditions (Fig. 4C, top). After differentiation, CSK-MSC derived from HS-expanded BM-MSC displayed a similar marker pattern to the other serum conditions, with positive staining for ALDH1A1, ALDH3A1, Lumican, and Keratocan, together with positive Collagen I staining, while α-SMA was not detected (Fig. 4C, bottom).

Together, these results indicate that BM-MSC expanded in FBS, hPL, or HS retain the ability to differentiate in 2D culture into cells with a CSK-like phenotype, showing positive staining of key keratocyte markers across all serum conditions.

### Serum alternatives promote transcriptional changes toward a keratocyte-like phenotype

To further assess the differentiation process, the relative gene expression of CSK-associated markers was quantified by qPCR before and after differentiation and normalized to BM-MSC cultured in FBS.

**Fig. 5:**
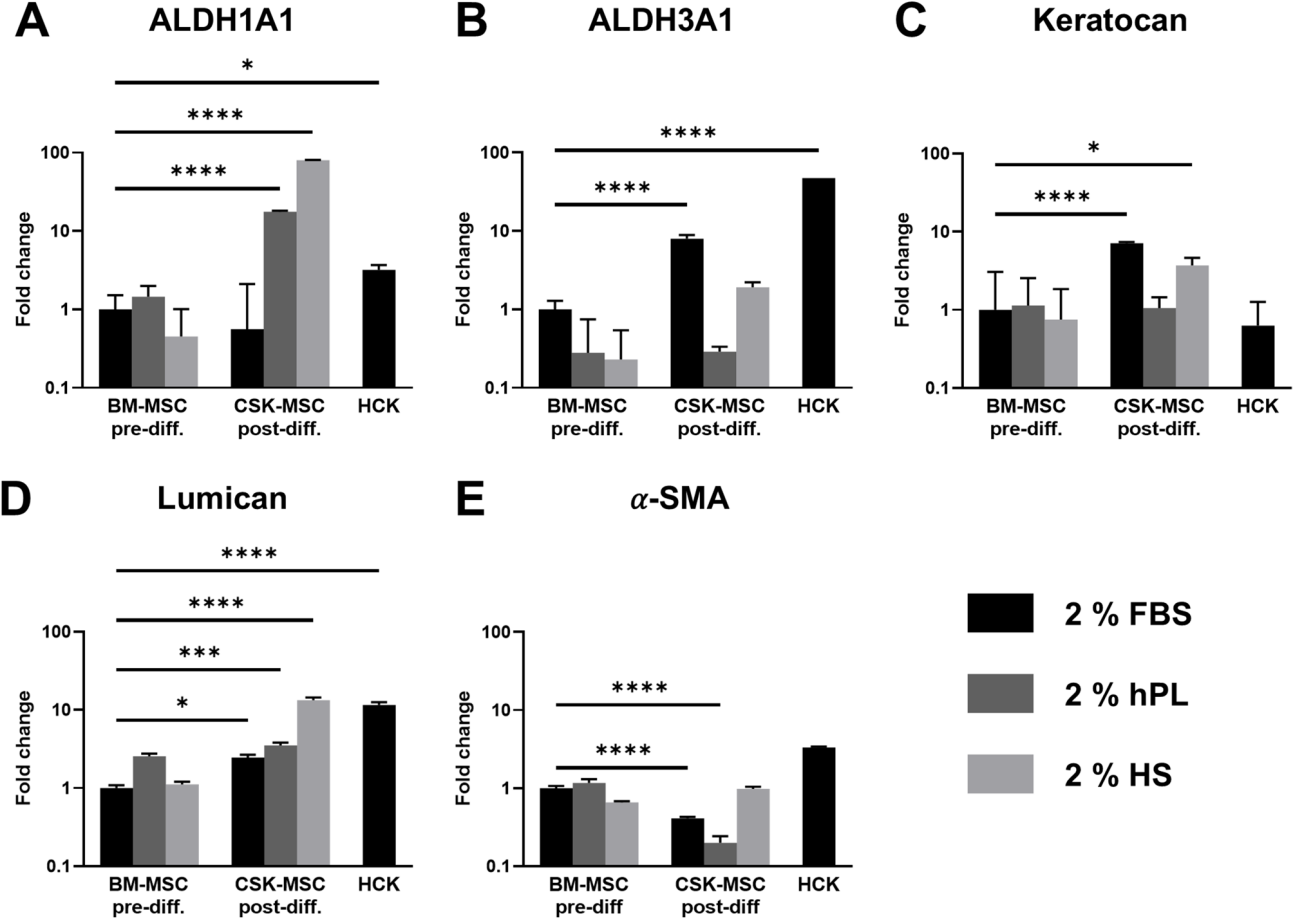
Comparative analysis of the expression of the CSK markers ALDH1A1 (A), ALDH3A1 (B), Keratocan (C), Lumican (D), and αSMA (E) before and after differentiation of BM-MSC in 2D. Expression was compared to human corneal keratocytes (HCK). The housekeeping gene Glyceraldehyde 3-phosphate dehydrogenase (GAPDH) was used as a reference and the expression in cells before differentiation cultivated in FBS was used for comparison. The fold change is shown on a logarithmic scale. Data are presented as mean ± standard deviation of three technical replicates (*n* = 3). * p-value ≤ 0.05, ** p-value ≤ 0.01, *** p-value ≤ 0.001, **** p-value ≤ 0.0001.

ALDH1A1 expression was significantly upregulated in CSK-MSC derived from cells cultivated in hPL (17.61 ± 0.48, *p* < 0.0001) and HS (80.23 ± 0.15, *p* < 0.0001), as well as in HCK (3.19 ± 0.49, *p* = 0.0122), compared with BM-MSC in FBS (Fig. 5A). Moreover, cells expanded in hPL and HS showed a further increase in ALDH1A1 expression after differentiation relative to their undifferentiated counterparts. The expression level in HCK was intermediate compared with CSK-MSC samples.

In contrast, ALDH3A1 was significantly upregulated in CSK-MSC cultivated in FBS before differentiation (7.95 ± 0.91, *p* < 0.0001) and strongly expressed in HCK (47.17 ± 0.08, *p* < 0.0001) compared with BM-MSC in FBS (Fig. 5B). An additional increase in ALDH3A1 expression was observed after differentiation in HS-cultured cells. Overall, HCK exhibited higher ALDH3A1 expression than all CSK-MSC groups.

Keratocan expression was significantly upregulated in CSK-MSC cultivated in FBS (7.16 ± 0.245, *p* < 0.0001) and HS (3.70 ± 0.93, *p* = 0.0215) after differentiation when compared with undifferentiated BM-MSC (Fig. 5C). The CSK-MSC in FBS was analyzed in a separate assay using the same housekeeping gene, ensuring consistent normalization. CSK-MSC generated in FBS, hPL or HS displayed comparable or higher Keratocan expression than HCK.

Lumican expression was significantly increased in all CSK-MSC conditions relative to BM-MSC in FBS (FBS: 2.46 ± 0.21, *p* = 0.0384; hPL: 3.51 ± 0.30, *p* = 0.0006; HS: 13.27 ± 1.12, *p* < 0.0001) (Fig. 5D). HCK also showed a strong upregulation of Lumican (11.56 ± 0.98, *p* < 0.0001). Overall expression levels in HCK and CSK-MSC were comparable.

The myofibroblast marker α-SMA was significantly downregulated in CSK-MSC derived from cell cultivated in FBS (0.041 ± 0.02, *p* < 0.0001) and hPL (0.20 ± 0.04, *p* < 0.0001) compared to BM-MSC in FBS. All differentiated conditions showed either downregulated or comparable α-SMA expression relative to HCK, consistent with the maintenance of a quiescent CSK-like phenotype (Fig. 5E).

### 3D RA-GelMA culture enables stable keratocyte-like differentiation

Immunofluorescence staining was performed to evaluate marker expression in 3D-encapsulated BM-MSC cultured in GelMA and supplemented with different serum alternatives before and after differentiation toward a CSK-like phenotype. Single signals for all immunofluorescence stainings in 3D are provided in the supplementary data (Figs. S7-S12).

**Fig. 6:**
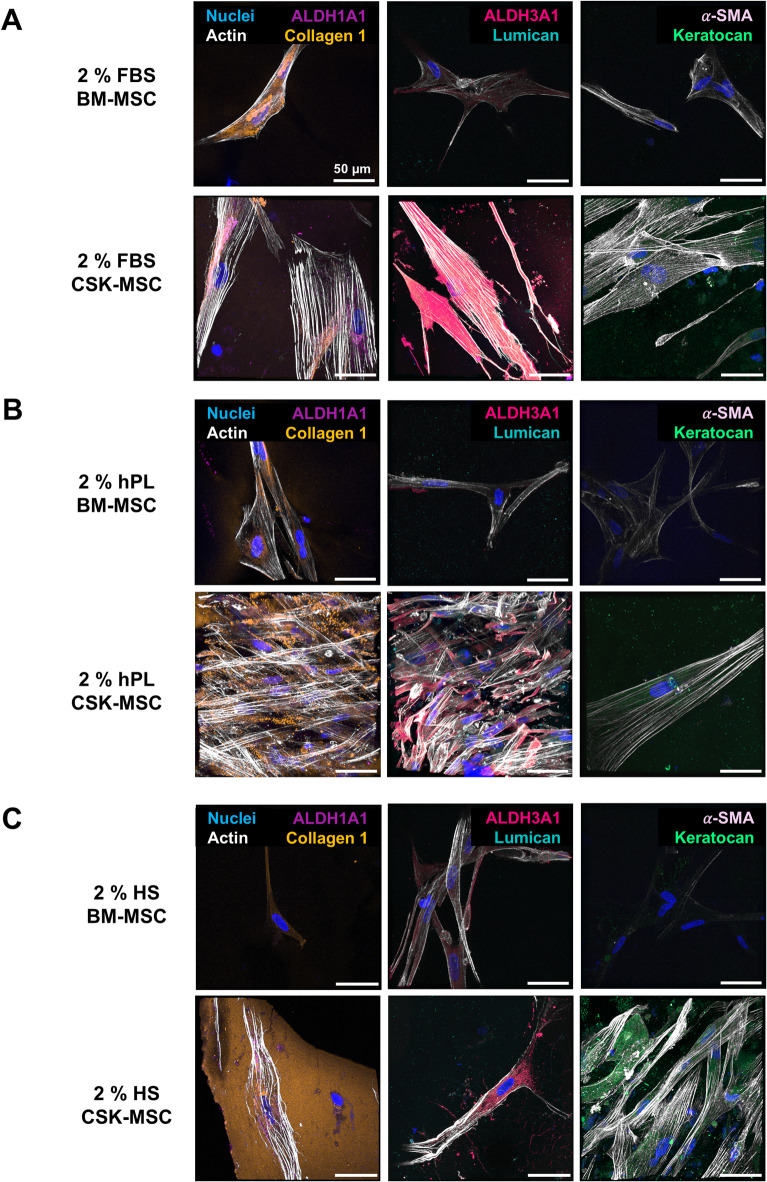
Immunostaining of 3D pooled BM-MSC and CSK-MSC encapsulated in 30 wt% RA-GelMA and cultured in Mesenpan supplemented with different serum conditions before and after differentiation. Cells were stained for ALDH1A1 (light pink), Collagen I (orange), ALDH3A1 (dark pink), Lumican (turquoise), α-SMA (lilac), and Keratocan (green) with Hoechst nuclear staining (blue) and phalloidin actin staining (white). Cells were encapsulated in 30 wt% RA-GelMA at a density of 2 × 10⁶ cells/mL. Scale bars represent 50 μm. Representative images from one technical replicate per antibody couple. All conditions were performed in three technical triplicates (*n* = 3). (A) Cells cultured with 2% FBS. Top: 3D BM-MSC before differentiation. Bottom: 3D CSK-MSC after differentiation. (B) Cells cultured with 2% hPL. Top: 3D BM-MSC before differentiation. Bottom: 3D CSK-MSC after differentiation. (C) Cells cultured with 2% HS. Top: 3D pooled BM-MSC before differentiation. Bottom: 3D CSK-MSC after differentiation.

Before differentiation, cells encapsulated in 30 wt% RA-GelMA and cultured in Mesenpan supplemented with 2% FBS showed detectable expression of ALDH1A1, ALDH3A1, and Collagen I, with detectable signal for Lumican (Fig. 6A, top). In contrast, Keratocan and α-SMA were not detected. After differentiation, CSK-MSC exhibited positive staining for the CSK-associated markers ALDH1A1, ALDH3A1, Collagen I, Lumican, and Keratocan (Fig. 6A, bottom), while α-SMA remained undetectable.

A comparable baseline marker profile was observed for 3D BM-MSC cultured with 2% hPL before differentiation, with detectable expression of ALDH1A1, ALDH3A1, Collagen I, and Lumican, and absence of Keratocan and α-SMA (Fig. 6B, top). After differentiation, CSK-MSC derived from hPL-expanded cells expressed ALDH1A1, ALDH3A1, Collagen I, Lumican, and Keratocan, while α-SMA expression remained absent (Fig. 6B, bottom).

For cells cultured in 3D with 2% HS, fluorescence signals for ALDH3A1, Collagen I, and Lumican were observed before differentiation, whereas ALDH1A1, Keratocan, and α-SMA were not detected (Fig. 6C, top). After differentiation, positive staining for all CSK markers, including ALDH1A1, ALDH3A1, Lumican, Keratocan, and Collagen I, were observed (Fig. 6C, bottom). Most cells remained negative for α-SMA, although localized signals were occasionally detected.

Overall, these results indicate that BM-MSC encapsulated in RA-GelMA and expanded with FBS, hPL, or HS retain the capacity to differentiate in 3D culture toward a CSK-like phenotype, characterized by the presence of keratocyte-associated markers and minimal expression of the myofibroblast marker α-SMA.

## Discussion

In this study, the effect of animal-free serum alternatives on BM-MSC metabolic activity and differentiation toward a CSK-like phenotype was evaluated in both 2D and 3D cultures. Overall, the results show that human-derived supplements, such as hPL and HS, can successfully support BM-MSC expansion and keratocyte differentiation, with performances comparable to conventional FBS conditions.

The XTT assay demonstrated a continuous increase in metabolic activity over time in all culture conditions, indicating good cell adaptation. At early time points, hPL and HS supported higher metabolic activity than FBS, suggesting that these human-derived supplements provide a favorable environment for BM-MSC growth, as previously reported for human platelet lysate and human serum-based culture systems^22,25,43^. A slight decrease in metabolic activity was observed in HS at later time points, this may reflect a stabilization phase following the initially enhanced metabolic activity observed at earlier time points, potentially associated with increasing cell density or adaptation to the serum environment. Importantly, all groups still showed a significant overall increase from day 1 to day 7, confirming that serum replacement did not impair cell survival.

Flow cytometry results showed that the type of serum influenced the MSC phenotype. BM-MSC cultured with 2% FBS exhibited a typical MSC marker profile, with positive expression of CD73, CD90, and CD105 and no expression of CD45, indicating that their mesenchymal identity was well preserved consistent with the minimal criteria defined by the International Society for Cellular Therapy^42^. A similar trend was observed in hPL and HS conditions, where cells expressed CD73 and CD105, with lower CD90 levels, and a population co-expressing the MSC markers without CD45 was detected. The slightly lower CD90 expression observed in BM-MSC may reflect the dynamic regulation of this marker during culture and may also be consistent with early stromal lineage commitment, as CD90 expression is generally reduced in quiescent CSK compared to mesenchymal progenitor cells^44,45^.

Immunofluorescence results in 2D culture showed that undifferentiated BM-MSC had low or absent expression of CSK markers, with only basal Collagen I and weak ALDH3A1 signals. After differentiation, cells cultured in FBS, hPL, or HS showed positive staining for the key keratocyte markers ALDH1A1, ALDH3A1, Lumican, and Keratocan, and Collagen I. Importantly, α-SMA was not detected following differentiation, indicating that the cells did not transition toward a myofibroblast phenotype, which is associated with fibrosis and loss of corneal transparency^46^.

These findings were confirmed at the gene level by qPCR analysis for the 2D cultures. Significant upregulation of ALDH1A1 and Lumican was observed after differentiation, particularly in hPL and HS conditions, with expression levels comparable to human corneal keratocytes. In parallel, α-SMA expression was strongly downregulated in differentiated samples, indicating the acquisition of a keratocyte-like phenotype rather than a fibrotic profile. These results are consistent with a previous report^47^ showing that hPL can support corneal stromal cell culture while preserving key phenotypic features, although its effect is concentration dependent. In particular, the presence of bioactive factors such as Hepatocyte growth factor and FGF-2 in hPL has been suggested to contribute to the suppression of myofibroblast differentiation^47^. In this context, the maintained or enhanced expression of stromal markers observed in our study, especially in hPL and HS conditions, may indicate that human-derived supplements provide a microenvironment that supports stromal lineage commitment while limiting fibrotic activation.

The 3D experiments in GelMA further supported the relevance of the model. Before differentiation, encapsulated BM-MSC showed detectable staining for ALDH1A1, ALDH3A1, Collagen I, and Lumican, while Keratocan and α-SMA were absent. After differentiation, positive staining for all primary CSK markers, including Keratocan, were observed across FBS, hPL, and HS conditions, with minimal or no α-SMA signal. This indicates that the 3D hydrogel environment supports keratocyte maturation while maintaining a non-fibrotic phenotype, highlighting the ability of 3D-based systems to support cell differentiation and matrix remodeling for corneal tissue engineering^7,35,37^.

From a translational perspective, replacing FBS with hPL or HS is highly relevant for the development of clinically applicable corneal models. Animal-derived serum is associated with batch variability, immunological risks, and regulatory limitations. In contrast, human-derived supplements are more suitable for the development of xeno-reduced culture systems and are better aligned with clinical and GMP requirements^40^. However, human-derived supplements may also present donor-to-donor and batch-to-batch variability, potentially influencing growth factor composition and cellular responses. The use of pooled donor products may help reduce this variability and improve reproducibility while maintaining the advantages of xeno-reduced culture systems^48–51^. Nevertheless, the comparable differentiation outcomes observed in all serum conditions suggest that FBS is not required to obtain a stable CSK-like phenotype post-differentiation.

The use of animal-free serum alternatives represents an important step toward clinically relevant systems^52^. A limitation of this study is the use of pooled BM-MSC from three donors, which prevented assessment of donor-to-donor variability. In addition, our current approach is not yet fully xeno-free due to the use of GelMA derived from bovine gelatin. As gelatin can be produced under GMP-compliant conditions and is already used clinically^53^, this provides a promising basis for future translation. In addition, the use of visible light crosslinking further supports the development of safer and more clinically compatible biofabrication strategies^54,55^. Building on this, future efforts should focus on transitioning toward fully defined or recombinant human-based hydrogels to establish completely xeno-free systems and further evaluate their suitability for translational corneal tissue engineering applications.

## Conclusion

BM-MSC cultured with hPL or HS retained high metabolic activity and efficiently differentiated into CSK-like cells in both 2D and 3D environments, with appropriate CSK marker expression and low myofibroblast activation. These findings demonstrate that human-derived serum alternatives can support the maintenance and differentiation of stromal cell phenotypes under xeno-reduced culture conditions. The established culture strategy represents a step toward more clinically relevant and xeno-reduced corneal tissue engineering approaches. These findings support future studies exploring the application of human-derived serum alternatives in corneal bioprinting, automated in situ repair models, and regenerative ophthalmology.

## Supplementary Information

Below is the link to the electronic supplementary material.

Supplementary Material 1

Supplementary Material 2

## Data Availability

The data that support the ﬁndings of this study are available from the corresponding author upon reasonable request.

## References

[1] Wang, E. Y. et al. Global trends in blindness and vision impairment resulting from corneal opacity 1984–2020: a meta-analysis. *Ophthalmology* **130**, 863–871. 10.1016/j.ophtha.2023.03.012 (2023). 10.1016/j.ophtha.2023.03.012 PMC10355344 36963570

[2] Lamm, V., Hara, H., Mammen, A., Dhaliwal, D. & Cooper, D. K. Corneal blindness and xenotransplantation. *Xenotransplantation* **21**, 99–114. 10.1111/xen.12082 (2014). 10.1111/xen.12082 PMC4181387 25268248

[3] Song, A. et al. Post-keratoplasty infectious keratitis: epidemiology, risk factors, management, and outcomes. *Front. Med. (Lausanne)*. **8**, 707242. 10.3389/fmed.2021.707242 (2021). 34307431 10.3389/fmed.2021.707242 PMC8292647

[4] Alio, J. L., Montesel, A., El Sayyad, F., Barraquer, R. I. & Arnalich-Montiel, F. Corneal graft failure: an update. *Br. J. Ophthalmol.* **105** (8), 1049–1058. 10.1136/bjophthalmol-2020-316705 (2021). 10.1136/bjophthalmol-2020-316705 32788325

[5] Gain, P. et al. Global survey of corneal transplantation and eye banking. *JAMA Ophthalmol.* **134** (2), 167–173. 10.1001/jamaophthalmol.2015.4776 (2016). 10.1001/jamaophthalmol.2015.4776 26633035

[6] AlMutlak, M., Li, J. Y., Helayel, H. B. & Fairaq, R. Future of corneal donation and transplantation: insights from the COVID-19 pandemic. *Cornea* **40**, 274–276. 10.1097/ICO.0000000000002538 (2021). 10.1097/ICO.0000000000002538 PMC7473794 32826649

[7] Duarte Campos, D. F. et al. Corneal bioprinting utilizing collagen-based bioinks and primary human keratocytes. *J. Biomed. Mater. Res. A*. **107** (9), 1945–1953. 10.1002/jbm.a.36702 (2019). 10.1002/jbm.a.36702 31012205

[8] Balters, L. & Reichl, S. 3D bioprinting of corneal models: a review of the current state and future outlook, *J. Tissue Eng.* **14**, 20417314231197793. 10.1177/20417314231197793 (2023). 10.1177/20417314231197793 PMC10504850 37719307

[9] Mobaraki, M. et al. Corneal repair and regeneration: current concepts and future directions. *Front. Bioeng. Biotechnol.* **7**, 135. 10.3389/fbioe.2019.00135 (2019). 31245365 10.3389/fbioe.2019.00135 PMC6579817

[10] Matthyssen, S., Van den Bogerd, B., Dhubhghaill, S. N., Koppen, C. & Zakaria, N. Corneal regeneration: a review of stromal replacements. *Acta Biomater.* **69**, 31–41. 10.1016/j.actbio.2018.01.023 (2018). 10.1016/j.actbio.2018.01.023 29374600

[11] Bonnet, C., González, S. & Deng, S. X. Ocular surface regeneration by limbal stem cells therapies: state of the art, challenges, and perspectives. *Stem Cells Transl. Med.* **12** (11), 714–719. 10.1093/stcltm/szad058 (2023). 37715946 10.1093/stcltm/szad058 PMC10630076

[12] Guérin, L. P. et al. The Human Tissue-Engineered Cornea (hTEC): recent progress. *Int. J. Mol. Sci.* **22**, 1291. https://www.mdpi.com/1422-0067/22/3/1291 (2021). 10.3390/ijms22031291 PMC7865732 33525484

[13] Wilson, S. E. Corneal myofibroblasts and fibrosis. *Exp. Eye Res.* **201**, 108272. 10.1016/j.exer.2020.108272 (2020). 10.1016/j.exer.2020.108272 PMC7736212 33010289

[14] Liu, S., Yang, W., Li, Y. & Sun, C. Fetal bovine serum, an important factor affecting the reproducibility of cell experiments. *Sci. Rep.* **13**, 2023. 10.1038/s41598-023-29060-7 (1942). 10.1038/s41598-023-29060-7 PMC9894865 36732616

[15] Delabie, W. et al. Comparison of culture media supplements identifies serum components in self-reported serum-free preparations. *Stem Cell. Res. Ther.* **16** (1), 454. 10.1186/s13287-025-04561-6 (2025). 10.1186/s13287-025-04561-6 PMC12382287 40859293

[16] Lee, D. Y. et al. Analysis of commercial fetal bovine serum (FBS) and its substitutes in the development of cultured meat. *Food Res. Int.* **174**, 113617. 10.1016/j.foodres.2023.113617 (2023). 10.1016/j.foodres.2023.113617 37986472

[17] Stival, A. C. S., da Silva, A. C. G. & Valadares, M. C. Qualitative and quantitative evaluation of Fetal Bovine Serum composition: toward ethical and best quality in vitro science. *NAM J.*10.1016/j.namjnl.2025.100047 (2025). 10.1016/j.namjnl.2025.100047 PMC13289150 42369414

[18] Aswad, H., Jalabert, A. & Rome, S. Depleting extracellular vesicles from fetal bovine serum alters proliferation and differentiation of skeletal muscle cells in vitro.*BMC Biotechnol.* **16**, 32. 10.1186/s12896-016-0262-0 (2016). 10.1186/s12896-016-0262-0 PMC4818850 27038912

[19] van der Valk, J. et al. Fetal Bovine Serum (FBS): past - present - future. *ALTEX* **35**, 99–118. 10.14573/altex.1705101 (2018). 10.14573/altex.1705101 28800376

[20] Gstraunthaler, G., Lindl, T. & van der Valk, J. A plea to reduce or replace fetal bovine serum in cell culture media. *Cytotechnology* **65**, 791–793. 10.1007/s10616-013-9633-8 (2013). 10.1007/s10616-013-9633-8 PMC3967615 23975256

[21] Weber, T. et al. Fetal bovine serum: how to leave it behind in the pursuit of more reliable science. *Front. Toxicol.* **7**, 1612903. 10.3389/ftox.2025.1612903 (2025). 40861932 10.3389/ftox.2025.1612903 PMC12371577

[22] Hemeda, H., Giebel, B. & Wagner, W. Evaluation of human platelet lysate versus fetal bovine serum for culture of mesenchymal stromal cells, *Cytotherapy* **16**, 170–180. 10.1016/j.jcyt.2013.11.004 (2014). 10.1016/j.jcyt.2013.11.004 24438898

[23] Subbiahanadar Chelladurai, K. et al. Alternative to FBS in animal cell culture - an overview and future perspective. *Heliyon* **7**, e07686. 10.1016/j.heliyon.2021.e07686 (2021). 10.1016/j.heliyon.2021.e07686 PMC8349753 34401573

[24] Romagnoli, L. et al. Cell-based medicinal products and the development of GMP-compliant processes and manufacturing. *BMC Proc.* **5**, 33. 10.1186/1753-6561-5-S8-O3 (2011). 10.1186/1753-6561-5-S8-O3 PMC3284923 22373285

[25] Burnouf, T., Strunk, D., Koh, M. B. & Schallmoser, K. Human platelet lysate: replacing fetal bovine serum as a gold standard for human cell propagation? *Biomaterials* **76**, 371–387. 10.1016/j.biomaterials.2015.10.065 (2016). 10.1016/j.biomaterials.2015.10.065 26561934

[26] Pytlik, R. et al. The cultivation of human multipotent mesenchymal stromal cells in clinical grade medium for bone tissue engineering. *Biomaterials* **30**, 3415–3427. 10.1016/j.biomaterials.2009.03.001 (2009). 10.1016/j.biomaterials.2009.03.001 19362364

[27] Dos Santos, V. T. et al. Characterization of human AB serum for mesenchymal stromal cell expansion. *Transfus. Med. Hemother*. **44** (1), 11–21. 10.1159/000448196 (2017). 10.1159/000448196 PMC5318935 28275329

[28] Guiotto, M., Raffoul, W., Hart, A. M., Riehle, M. O. & di Summa, P. G. Human platelet lysate to substitute fetal bovine serum in hMSC expansion for translational applications: a systematic review. *J. Transl. Med.* **18** (1), 351. 10.1186/s12967-020-02489-4 (2020). 10.1186/s12967-020-02489-4 PMC7493356 32933520

[29] Psychogios, N. et al. The human serum metabolome. *PLoS One* **6**, e16957. 10.1371/journal.pone.0016957 (2011). 10.1371/journal.pone.0016957 PMC3040193 21359215

[30] Lange, C. et al. Accelerated and safe expansion of human mesenchymal stromal cells in animal serum-free medium for transplantation and regenerative medicine. *J. Cell. Physiol.* **213** (1), 18–26. 10.1002/jcp.21081 (2007). 10.1002/jcp.21081 17458897

[31] Doucet, C. et al. Platelet lysates promote mesenchymal stem cell expansion: a safety substitute for animal serum in cell-based therapy applications. *J. Cell Physiol.* **205**, 228–236. 10.1002/jcp.20391 (2005). 10.1002/jcp.20391 15887229

[32] Han, Y. et al. Mesenchymal stem cells for regenerative medicine. *Cells* **8**, 13. 10.3390/cells8080886 (2019). 10.3390/cells8080886 PMC6721852 31412678

[33] Neo, S. H. et al. Expansion of human bone marrow-derived mesenchymal stromal cells with enhanced immunomodulatory properties. *Stem Cell. Res. Ther.* **14** (1), 259. 10.1186/s13287-023-03481-7 (2023). 10.1186/s13287-023-03481-7 PMC10510228 37726837

[34] Dos Santos, A. et al. Differentiation capacity of human mesenchymal stem cells into keratocyte lineage. *Invest. Ophthalmol. Vis. Sci.* **60** (8), 3013–3023. 10.1167/iovs.19-27008 (2019). 10.1167/iovs.19-27008 PMC6636549 31310658

[35] Taoum, A. et al. 3D differentiation of bone-marrow derived mesenchymal stromal cells into the keratocyte lineage for corneal bioprinting. *Adv. Healthc. Mater.* **14** (28), e2405073. 10.1002/adhm.202405073 (2025). 10.1002/adhm.202405073 PMC12581885 40734361

[36] Piao, Y. et al. Biomedical applications of gelatin methacryloyl hydrogels. *Eng. Regener.* **2**, 47–56. 10.1016/j.engreg.2021.03.002 (2021).

[37] Dehli, F. et al. Biobased photocrosslinkable gelatin-methacrylate hydrogels promote the growth and phenotype maintenance of human corneal keratocytes. *Mater. Adv.* **6** (12), 3805–3816. 10.1039/d5ma00076a (2025).

[38] Nishida, T. Commanding roles of keratocytes in health and disease. *Cornea* **29**, S3–6. 10.1097/ICO.0b013e3181f2d578 (2010). 10.1097/ICO.0b013e3181f2d578 20935539

[39] Yam, G. H. F., Riau, A. K., Funderburgh, M. L., Mehta, J. S. & Jhanji, V. Keratocyte biology. *Exp. Eye Res.* **196**, 108062. 10.1016/j.exer.2020.108062 (2020). 10.1016/j.exer.2020.108062 32442558

[40] van der Valk, J. et al. Optimization of chemically defined cell culture media–replacing fetal bovine serum in mammalian in vitro methods. *Toxicol. Vitro*. **24** (4), 1053–1063. 10.1016/j.tiv.2010.03.016 (2010). 10.1016/j.tiv.2010.03.016 20362047

[41] Claassen, C., Southan, A., Grubel, J., Tovar, G. E. M. & Borchers, K. Interactions of methacryloylated gelatin and heparin modulate physico-chemical properties of hydrogels and release of vascular endothelial growth factor. *Biomed. Mater.* **13** (5), 055008. 10.1088/1748-605X/aacdb2 (2018). 10.1088/1748-605X/aacdb2 29923498

[42] Dominici, M. et al. Minimal criteria for defining multipotent mesenchymal stromal cells. The International Society for Cellular Therapy position statement. *Cytotherapy* **8**, 315–317. 10.1080/14653240600855905 (2006). 10.1080/14653240600855905 16923606

[43] Kolesnikova, I. S. et al. Effect of platelet lysate on mammalian cell proliferation. *J. Evol. Biochem. Physiol.* **62** (1), 70–78. 10.1134/s0022093026010072 (2026).

[44] Pei, Y., Sherry, D. M. & McDermott, A. M. Thy-1 distinguishes human corneal fibroblasts and myofibroblasts from keratocytes. *Exp. Eye Res.* **79** (5), 705–712. 10.1016/j.exer.2004.08.002 (2004). 10.1016/j.exer.2004.08.002 15500828

[45] Sidney, L. E. & Hopkinson, A. Corneal keratocyte transition to mesenchymal stem cell phenotype and reversal using serum-free medium supplemented with fibroblast growth factor-2, transforming growth factor-beta3 and retinoic acid. *J. Tissue Eng. Regen Med.* **12** (1), e203–e215. 10.1002/term.2316 (018). 10.1002/term.2316 27685949

[46] Guo, X., Sriram, S., Tran, J. A., Hutcheon, A. E. K. & Zieske, J. D. Inhibition of human corneal myofibroblast formation. *Invest. Ophthalmol. Vis. Sci.* **59** (8), 3511–3520. 10.1167/iovs.18-24239 2018). 10.1167/iovs.18-24239 PMC6044211 30025094

[47] Seidelmann, N. et al. Human platelet lysate as a replacement for fetal bovine serum in human corneal stromal keratocyte and fibroblast culture. *J. Cell. Mol. Med.* **25** (20), 9647–9659. 10.1111/jcmm.16912 (2021). 10.1111/jcmm.16912 PMC8505853 34486211

[48] dos Santos, V. T. M. et al. Characterization of human AB serum for mesenchymal stromal cell expansion. *Transfus. Med. Hemotherapy*. **44** (1), 11–21. 10.1159/000448196 (2016). 10.1159/000448196 PMC5318935 28275329

[49] Fernandez-Rebollo, E. et al. Human platelet lysate versus fetal calf serum: these supplements do not select for different mesenchymal stromal cells. *Sci. Rep.* **7**, 5132. 10.1038/s41598-017-05207-1 (2017). 10.1038/s41598-017-05207-1 PMC5506010 28698620

[50] Schallmoser, K., Henschler, R., Gabriel, C., Koh, M. B. C. & Burnouf, T. Production and quality requirements of human platelet lysate: a position statement from the working party on cellular therapies of the international society of blood transfusion. *Trends Biotechnol.* **38** (1), 13–23. 10.1016/j.tibtech.2019.06.002 (2020). 10.1016/j.tibtech.2019.06.002 31326128

[51] Bieback, K., Fernandez-Munoz, B., Pati, S. & Schafer, R. Gaps in the knowledge of human platelet lysate as a cell culture supplement for cell therapy: a joint publication from the AABB and the International Society for Cell & Gene Therapy. *Cytotherapy* **21**, 911–924. 10.1016/j.jcyt.2019.06.006 (2019). 10.1016/j.jcyt.2019.06.006 31307904

[52] Jian, Y., Dehli, F., Wisbar, M., Taoum, A. & Duarte Campos, D. In situbioprinting: bioprinting methods, bioinks, cell sources & advanced bioprinting strategies. *Biofabrication* **18**, 1. 10.1088/1758-5090/ae3cc3 (2026). 10.1088/1758-5090/ae3cc3 41582830

[53] Klotz, B. J., Gawlitta, D., Rosenberg, A., Malda, J. & Melchels, F. P. W. Gelatin-methacryloyl hydrogels: towards biofabrication-based tissue repair. *Trends Biotechnol.* **34** (5), 394–407. 10.1016/j.tibtech.2016.01.002 (2016). 10.1016/j.tibtech.2016.01.002 PMC5937681 26867787

[54] Sharifi, S., Sharifi, H., Akbari, A. & Chodosh, J. Systematic optimization of visible light-induced crosslinking conditions of gelatin methacryloyl (GelMA). *Sci. Rep.* **11** (1), 23276. 10.1038/s41598-021-02830-x (2021). 10.1038/s41598-021-02830-x PMC8640009 34857867

[55] Liao, Y. et al. A bioequivalent cornea cross-linking method using photo-initiators LAP and visible light. *Mater. Today Bio* **34**, 102110. 10.1016/j.mtbio.2025.102110 (2025). 10.1016/j.mtbio.2025.102110 PMC12318293 40755901

