# Supplementary material for: Toward clinically relevant automated corneal biomanufacturing with human-derived FBS alternatives
Source: Sci Rep. 2026 Jun 17;16:18874. doi: 10.1038/s41598-026-58401-5 (PMC13276165; doi:10.1038/s41598-026-58401-5)
Supplement: Supplementary file 1 — Supplementary Material 1 [file 41598_2026_58401_MOESM1_ESM.pdf]

Supplementary material

Toward Clinically Relevant Automated Corneal Biomanufacturing with Human-Derived FBS Alternatives

Alexandre Taoum[1,*], Julia S. Oster[1], Ole Thaden[1], Andrea Frank[1], Meng Wang[1], Mario Wisbar[1], Matthias Fuest[2], Friederike Dehli[1], Daniela Duarte Campos[1,*]

[1] Bioprinting & Tissue Engineering Group, Center for Molecular Biology of Heidelberg University [2] Department of Ophthalmology, RWTH Aachen University
*Correspondence to: a.taoum@zmbh.uni-heidelberg.de; dcampos@uni-heidelberg.de

This Supplementary information contains:

- Figure S1
- Figure S2
- Figure S3
- Figure S4
- Figure S5
- Figure S6
- Figure S7
- Figure S8
- Figure S9
- Figure S10
- Figure S11
- Figure S12

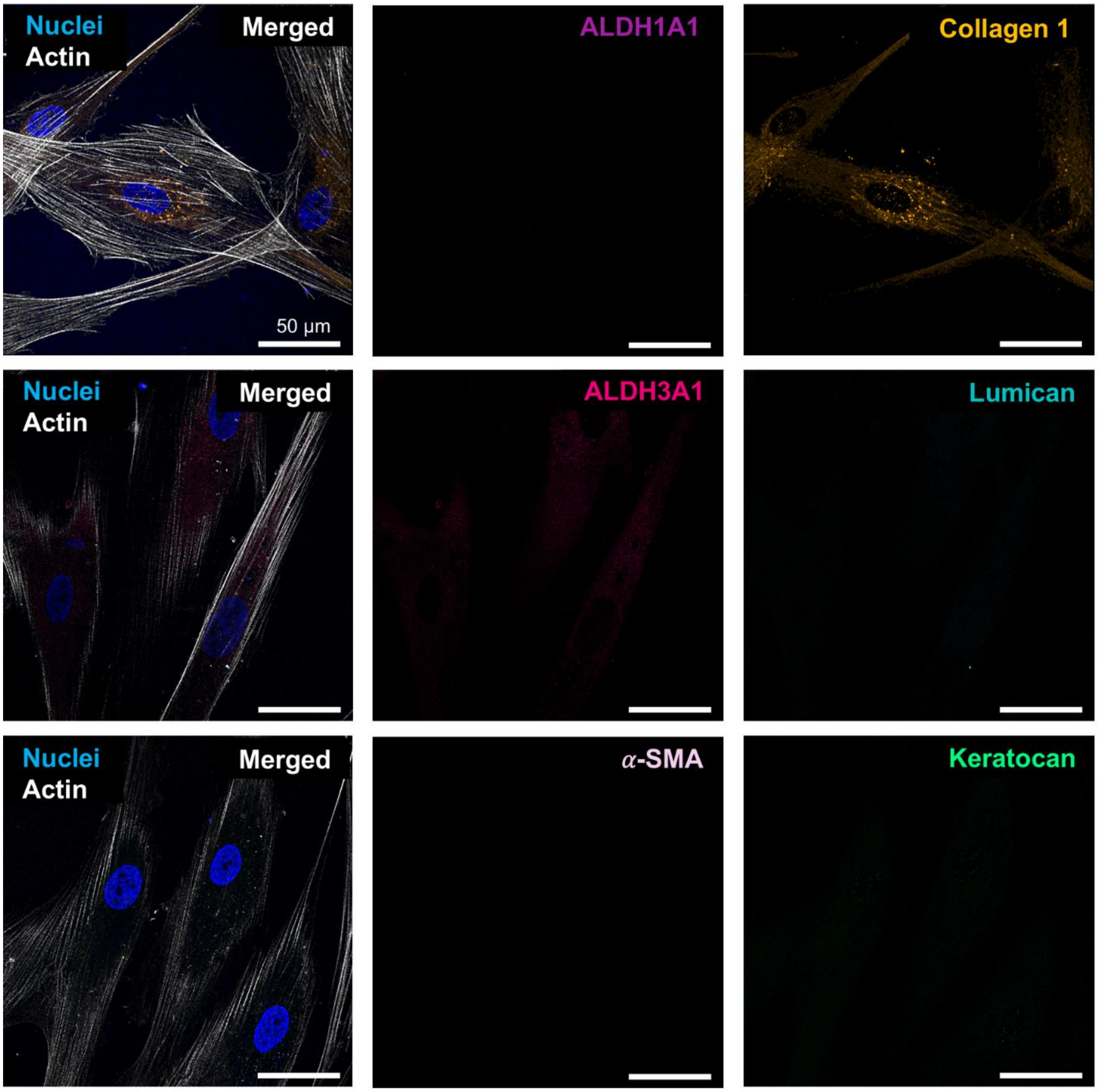

Figure S1. Immunostaining of 2D BM-MSC cultivated in Mesenpan with 2 % FBS before differentiation for ALDH1A1 (light pink), Collagen 1 (orange), ALDH3A1 (dark pink), Lumican (turquoise), α-SMA (lilac), and Keratocan (green) with Hoechst Nuclear Staining (blue) and phalloidin actin staining (white). Representative images from one technical replicate per antibody couple. All conditions were performed in three technical triplicates (n = 3).

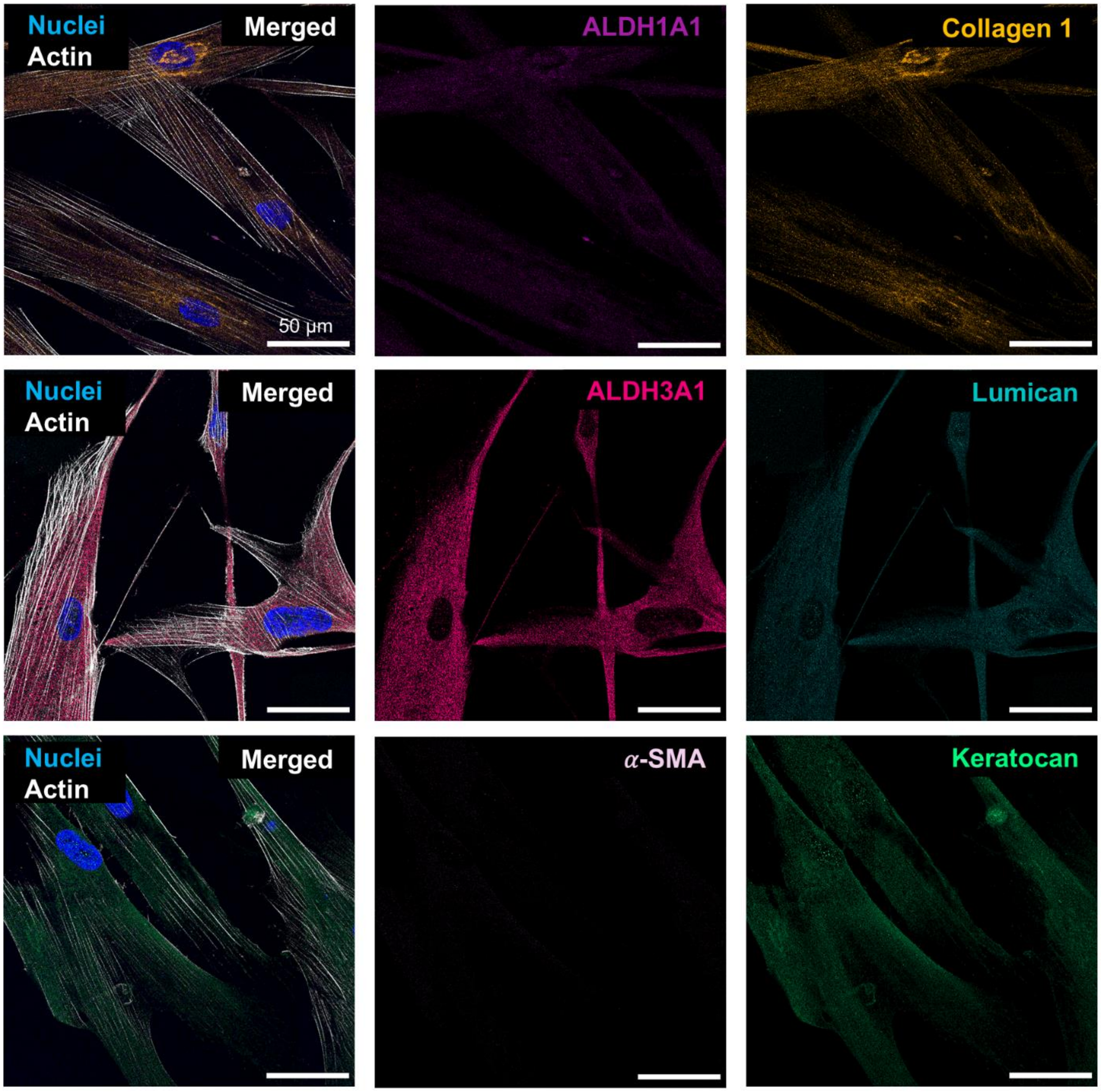

Figure S2. Immunostaining of 2D MSC-CSK derived from BM-MSC cultivated in Mesenpan with 2 % FBS after differentiation for ALDH1A1 (light pink), Collagen 1 (orange), ALDH3A1 (dark pink), Lumican (turquoise), α-SMA (lilac), and Keratocan (green) with Hoechst Nuclear Staining (blue) and phalloidin actin staining (white). Representative images from one technical replicate per antibody couple. All conditions were performed in three technical triplicates (n = 3).

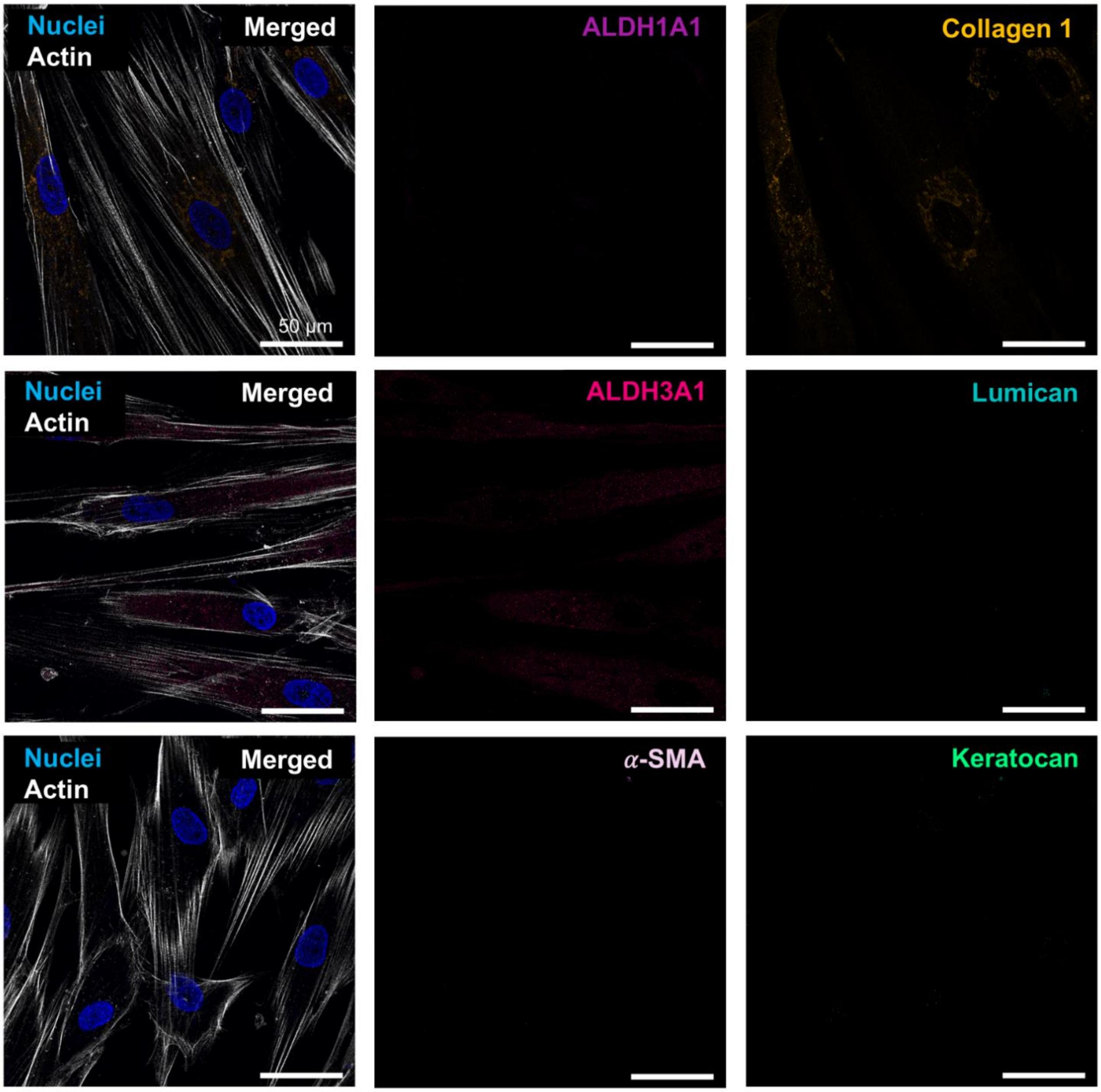

Figure S3. Immunostaining of 2D BM-MSC cultivated in Mesenpan with 2 % hPL before differentiation for ALDH1A1 (light pink), Collagen 1 (orange), ALDH3A1 (dark pink), Lumican (turquoise), α-SMA (lilac), and Keratocan (green) with Hoechst Nuclear Staining (blue) and phalloidin actin staining (white). Representative images from one technical replicate per antibody couple. All conditions were performed in three technical triplicates (n = 3).

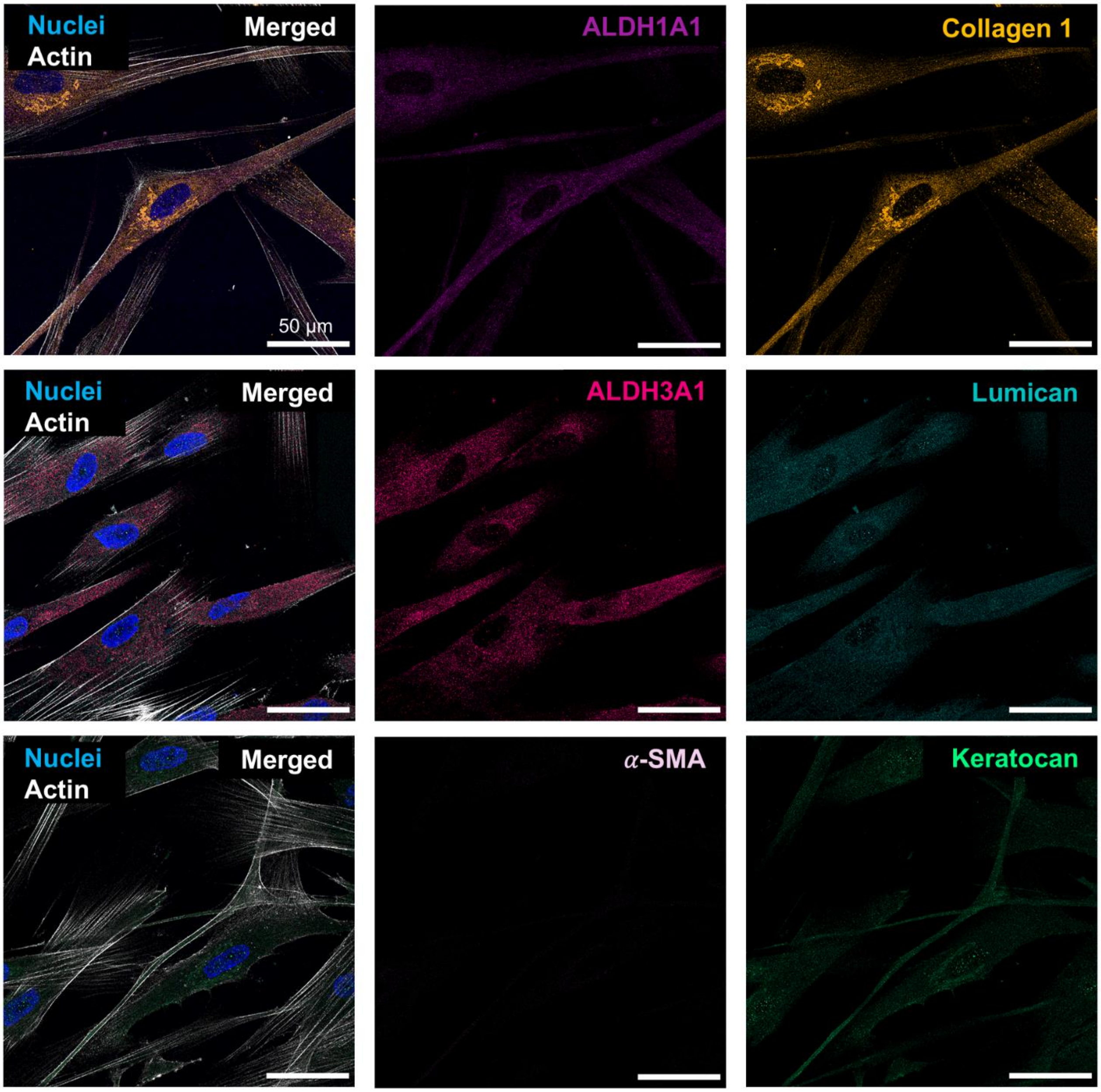

Figure S4. Immunostaining of 2D MSC-CSK derived from BM-MSC cultivated in Mesenpan with 2 % hPL after differentiation for ALDH1A1 (light pink), Collagen 1 (orange), ALDH3A1 (dark pink), Lumican (turquoise), α-SMA (lilac), and Keratocan (green) with Hoechst Nuclear Staining (blue) and phalloidin actin staining (white). Representative images from one technical replicate per antibody couple. All conditions were performed in three technical triplicates (n = 3).

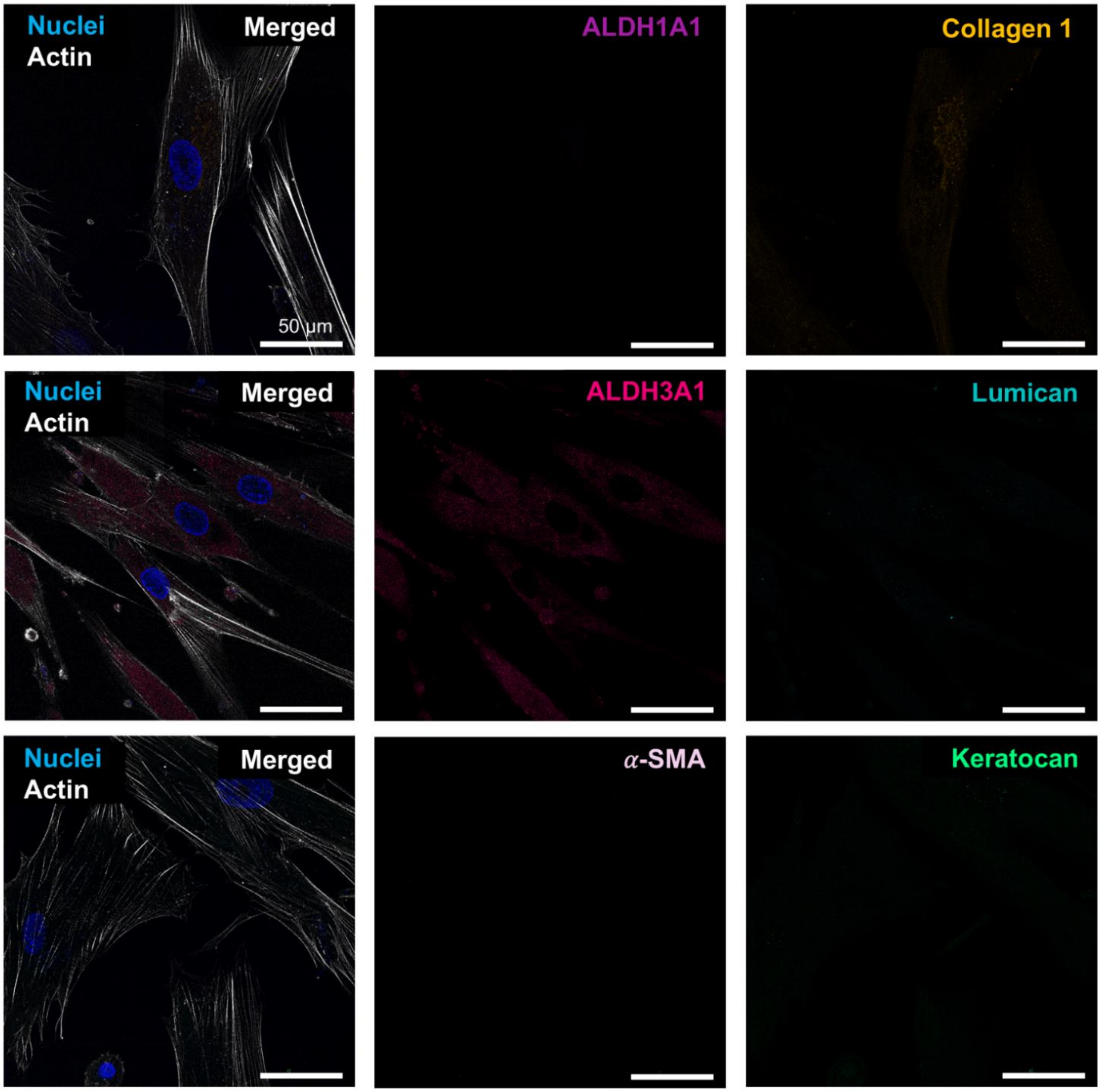

Figure S5. Immunostaining of 2D BM-MSC cultivated in Mesenpan with 2 % HS before differentiation for ALDH1A1 (light pink), Collagen 1 (orange), ALDH3A1 (dark pink), Lumican (turquoise), α-SMA (lilac), and Keratocan (green) with Hoechst Nuclear Staining (blue) and phalloidin actin staining (white). Representative images from one technical replicate per antibody couple. All conditions were performed in three technical triplicates (n = 3).

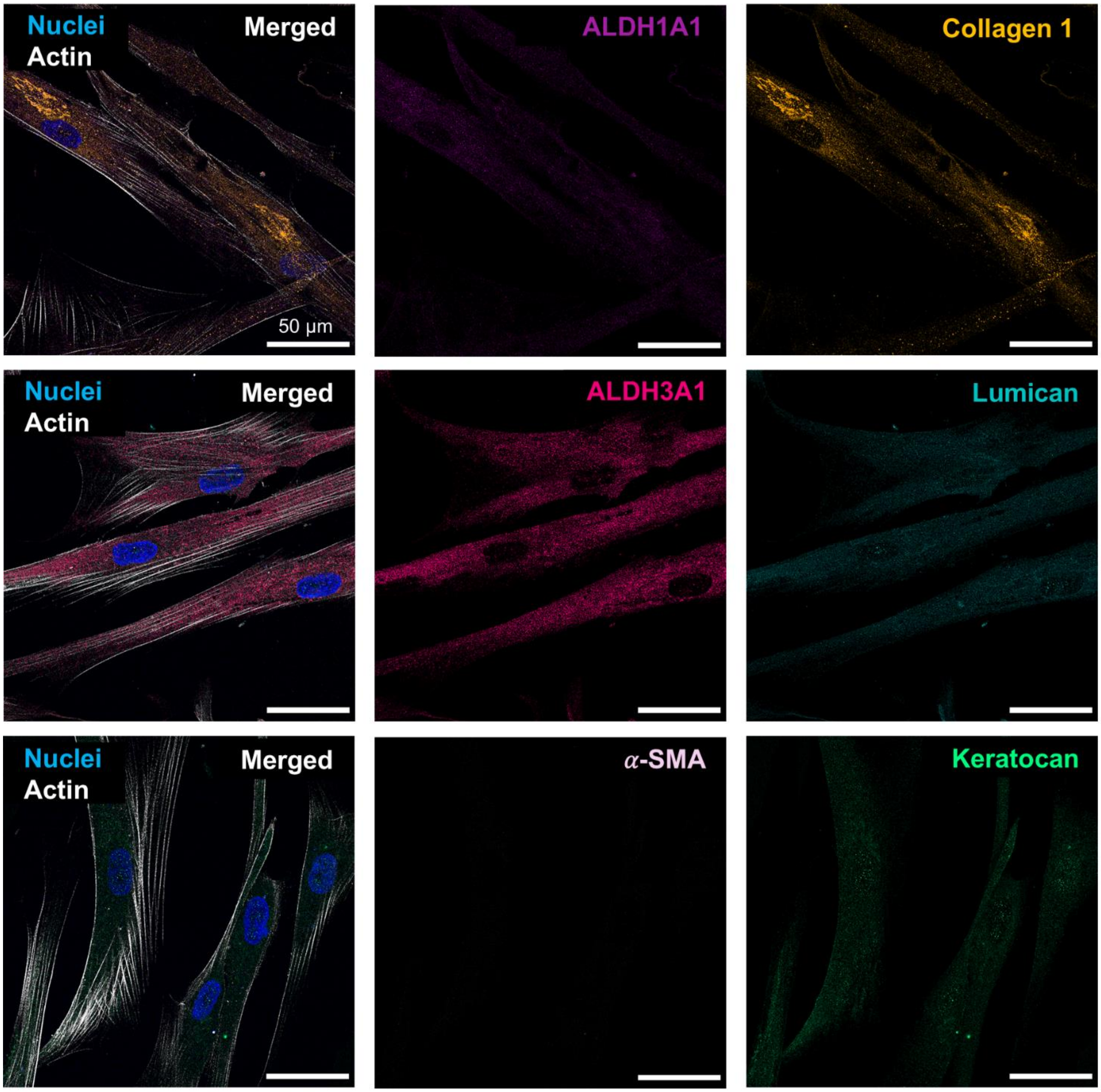

Figure S6. Immunostaining of 2D pooled MSC-CSK derived from BM-MSC cultivated in Mesenpan with 2 % HS after differentiation for ALDH1A1 (light pink), Collagen 1 (orange), ALDH3A1 (dark pink), Lumican (turquoise), α-SMA (lilac), and Keratocan (green) with Hoechst Nuclear Staining (blue) and phalloidin actin staining (white). Representative images from one technical replicate per antibody couple. All conditions were performed in three technical triplicates (n = 3).

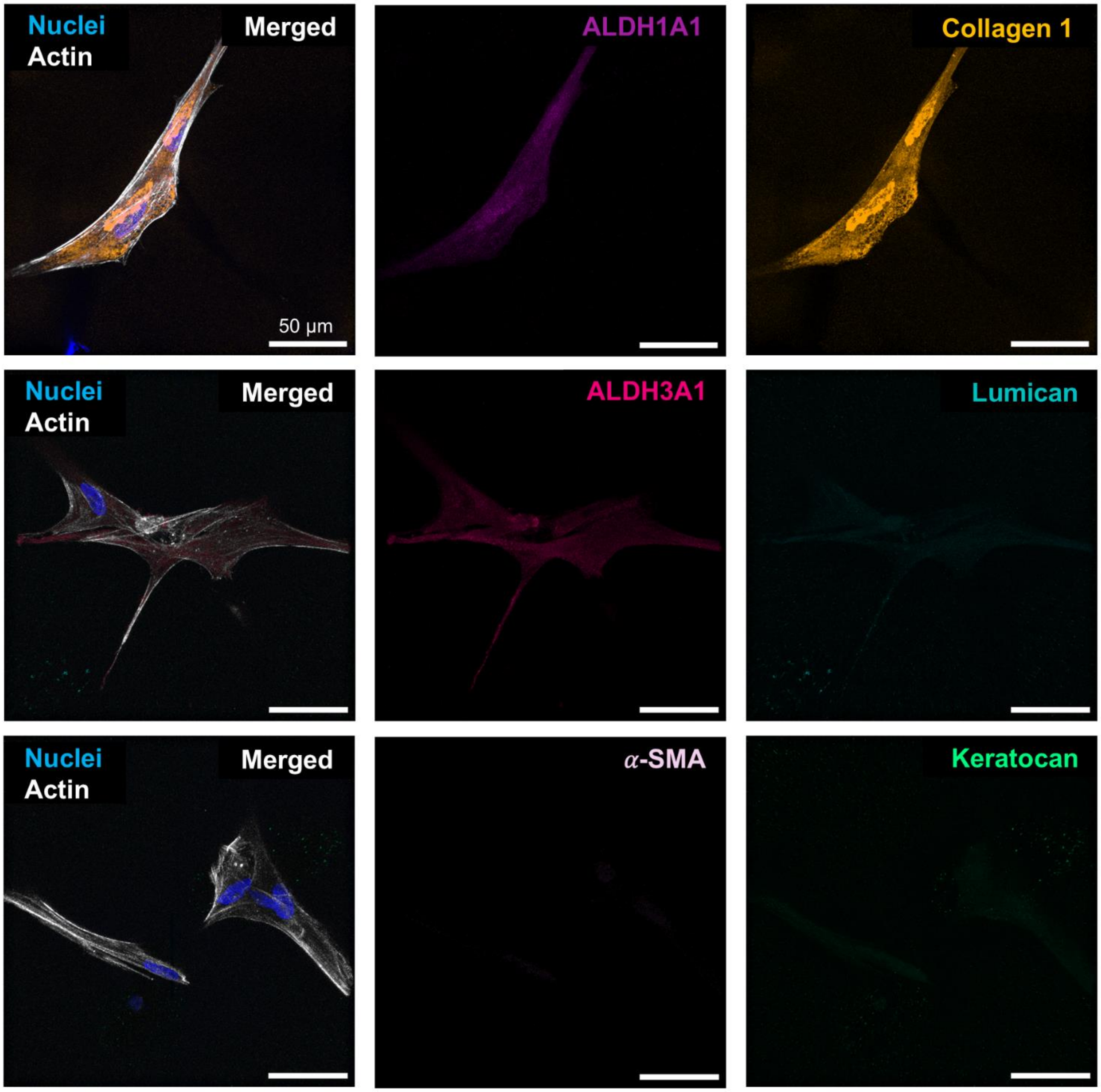

Figure S7. Immunostaining of 3D BM-MSC encapsulated in 30 wt % RA-GelMA cultivated in Mesenpan with 2 % FBS before differentiation for ALDH1A1 (light pink), Collagen 1 (orange), ALDH3A1 (dark pink), Lumican (turquoise), α-SMA (lilac), and Keratocan (green) with Hoechst Nuclear Staining (blue) and phalloidin actin staining (white). Representative images from one technical replicate per antibody couple. All conditions were performed in three technical triplicates (n = 3).

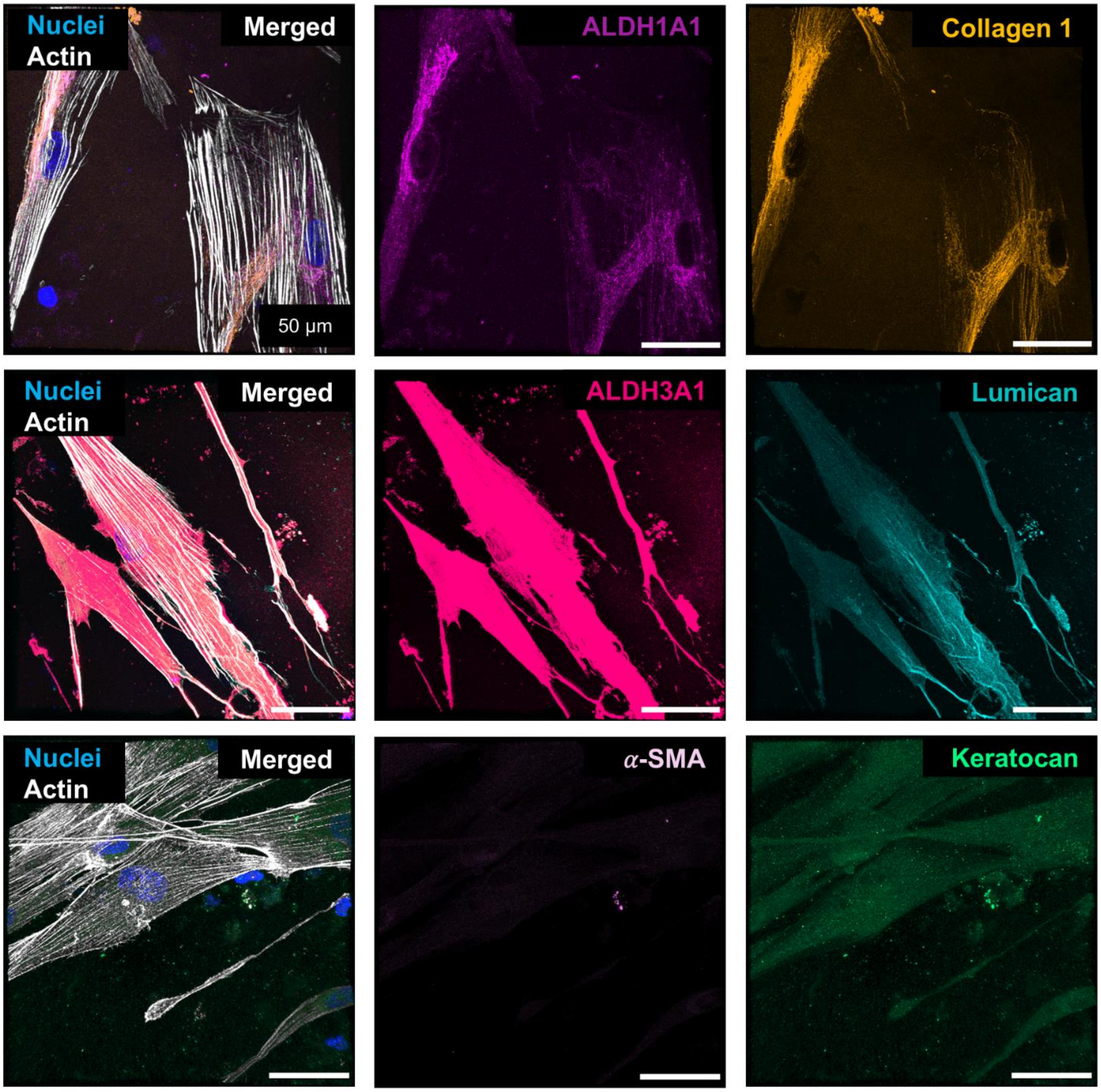

Figure S8. Immunostaining of 3D CSK-MSC encapsulated in 30 wt % RA-GelMA cultivated in Mesenpan with 2 % FBS after differentiation for ALDH1A1 (light pink), Collagen 1 (orange), ALDH3A1 (dark pink), Lumican (turquoise), α-SMA (lilac), and Keratocan (green) with Hoechst Nuclear Staining (blue) and phalloidin actin staining (white). Representative images from one technical replicate per antibody couple. All conditions were performed in three technical triplicates (n = 3).

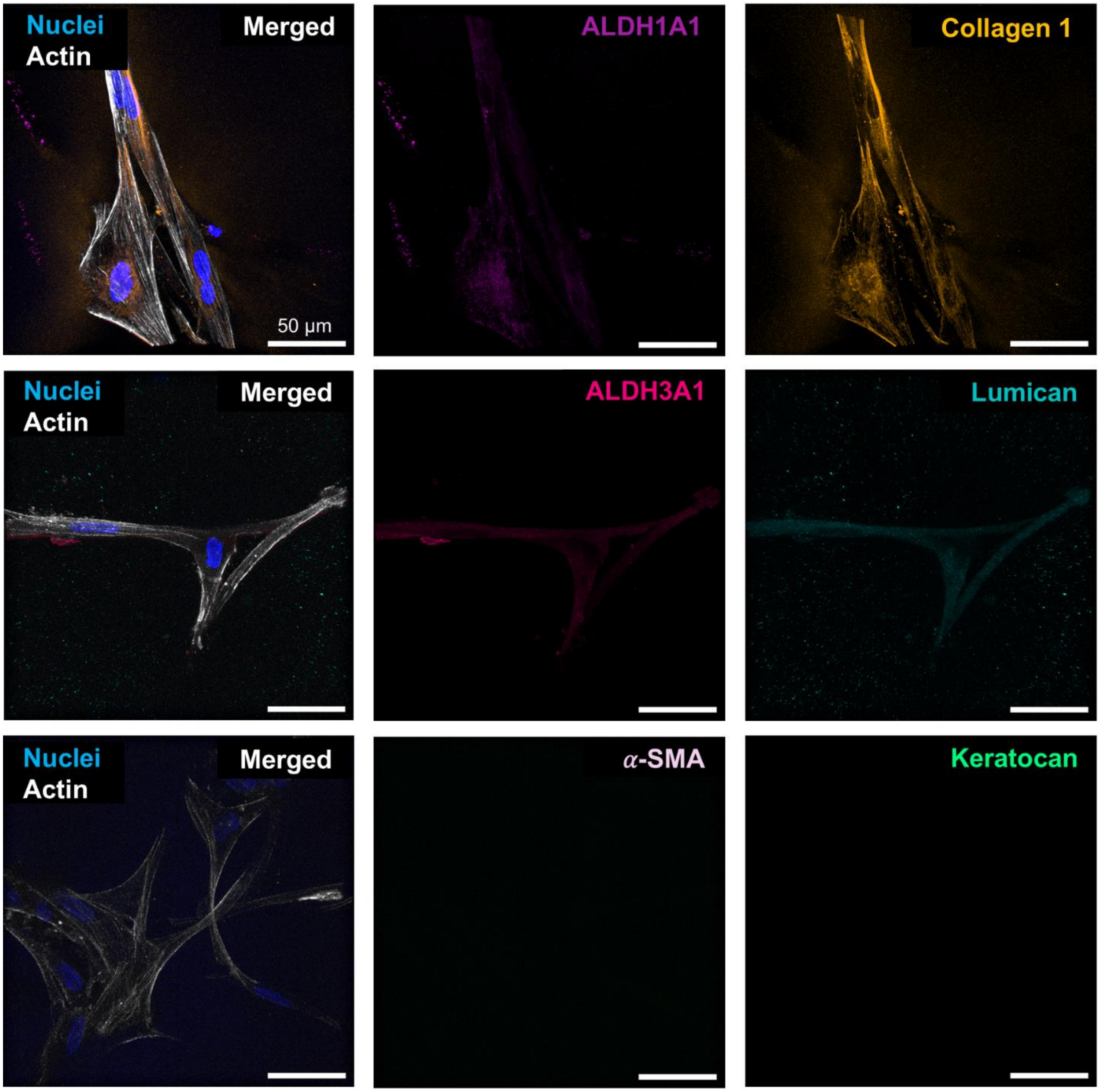

Figure S9. Immunostaining of 3D BM-MSC encapsulated in 30 wt % RA-GelMA cultivated in Mesenpan with 2 % hPL before differentiation for ALDH1A1 (light pink), Collagen 1 (orange), ALDH3A1 (dark pink), Lumican (turquoise), α-SMA (lilac), and Keratocan (green) with Hoechst Nuclear Staining (blue) and phalloidin actin staining (white). Representative images from one technical replicate per antibody couple. All conditions were performed in three technical triplicates (n = 3).

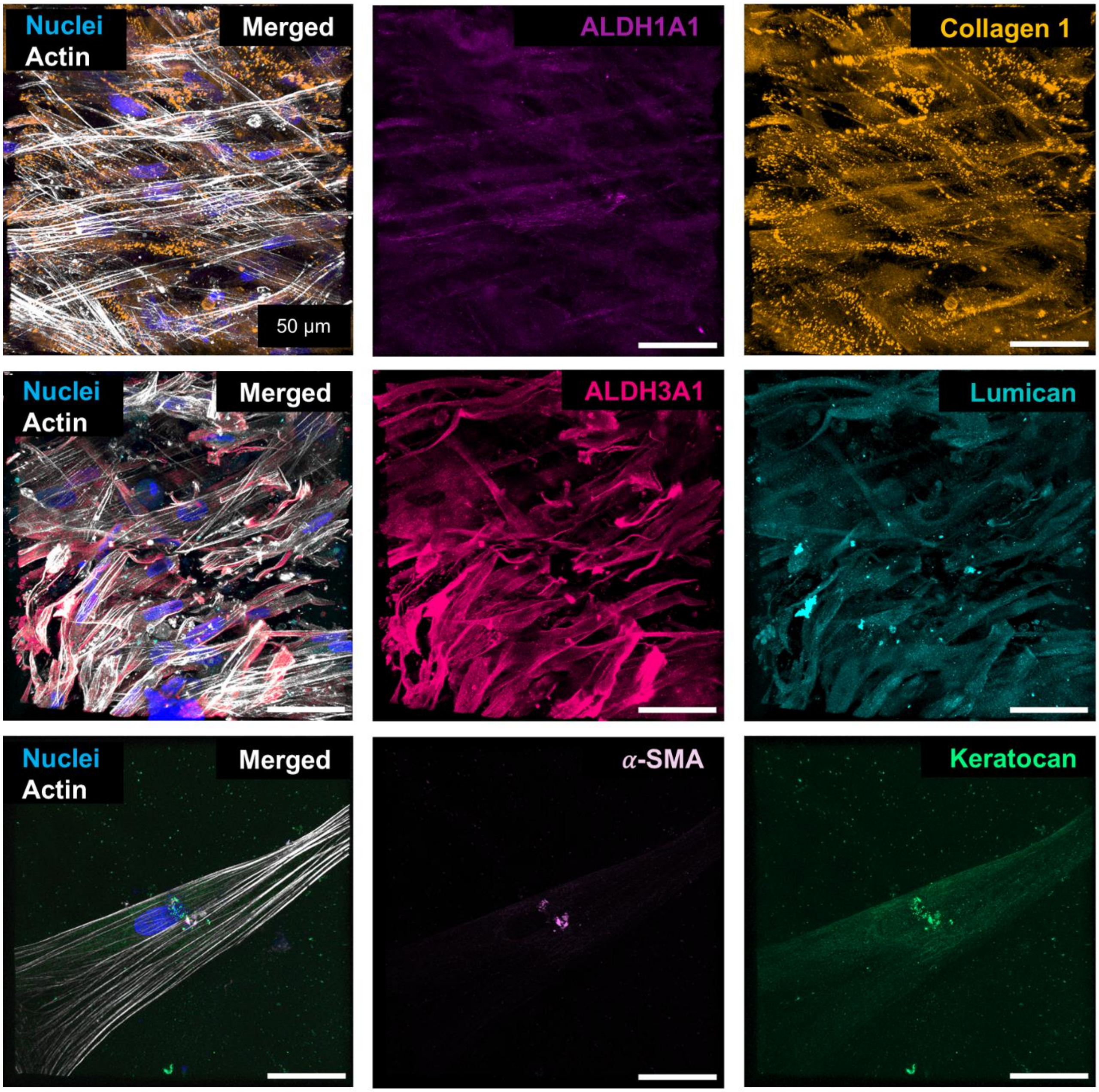

Figure S10. Immunostaining of 3D CSK-MSC encapsulated in 30 wt % RA-GelMA cultivated in Mesenpan with 2 % hPL after differentiation for ALDH1A1 (light pink), Collagen 1 (orange), ALDH3A1 (dark pink), Lumican (turquoise), α-SMA (lilac), and Keratocan (green) with Hoechst Nuclear Staining (blue) and phalloidin actin staining (white). Representative images from one technical replicate per antibody couple. All conditions were performed in three technical triplicates (n = 3).

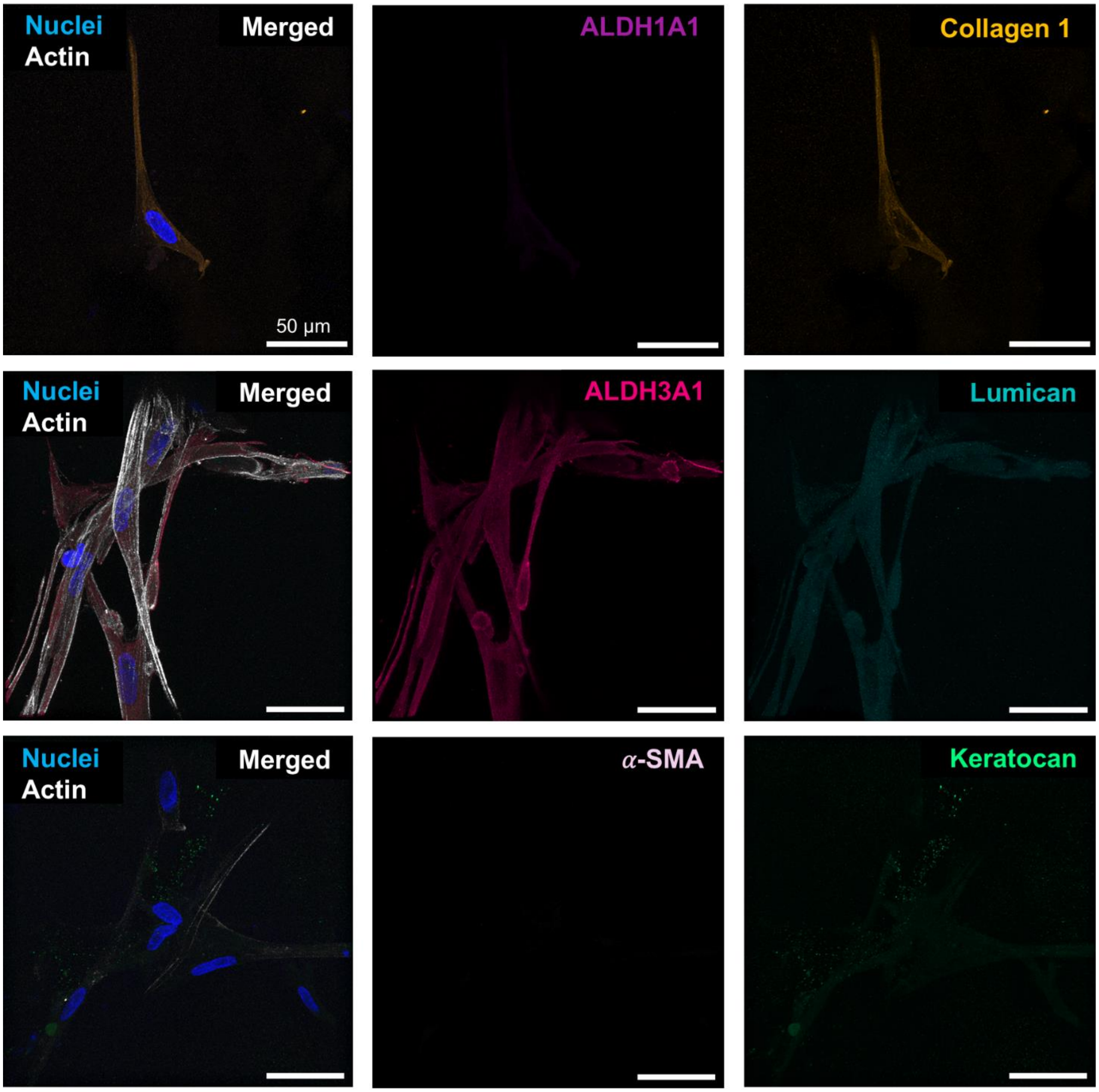

Figure S11. Immunostaining of 3D BM-MSC encapsulated in 30 wt % RA-GelMA cultivated in Mesenpan with 2 % HS before differentiation for ALDH1A1 (light pink), Collagen 1 (orange), ALDH3A1 (dark pink), Lumican (turquoise), α-SMA (lilac), and Keratocan (green) with Hoechst Nuclear Staining (blue) and phalloidin actin staining (white). Representative images from one technical replicate per antibody couple. All conditions were performed in three technical triplicates (n = 3).

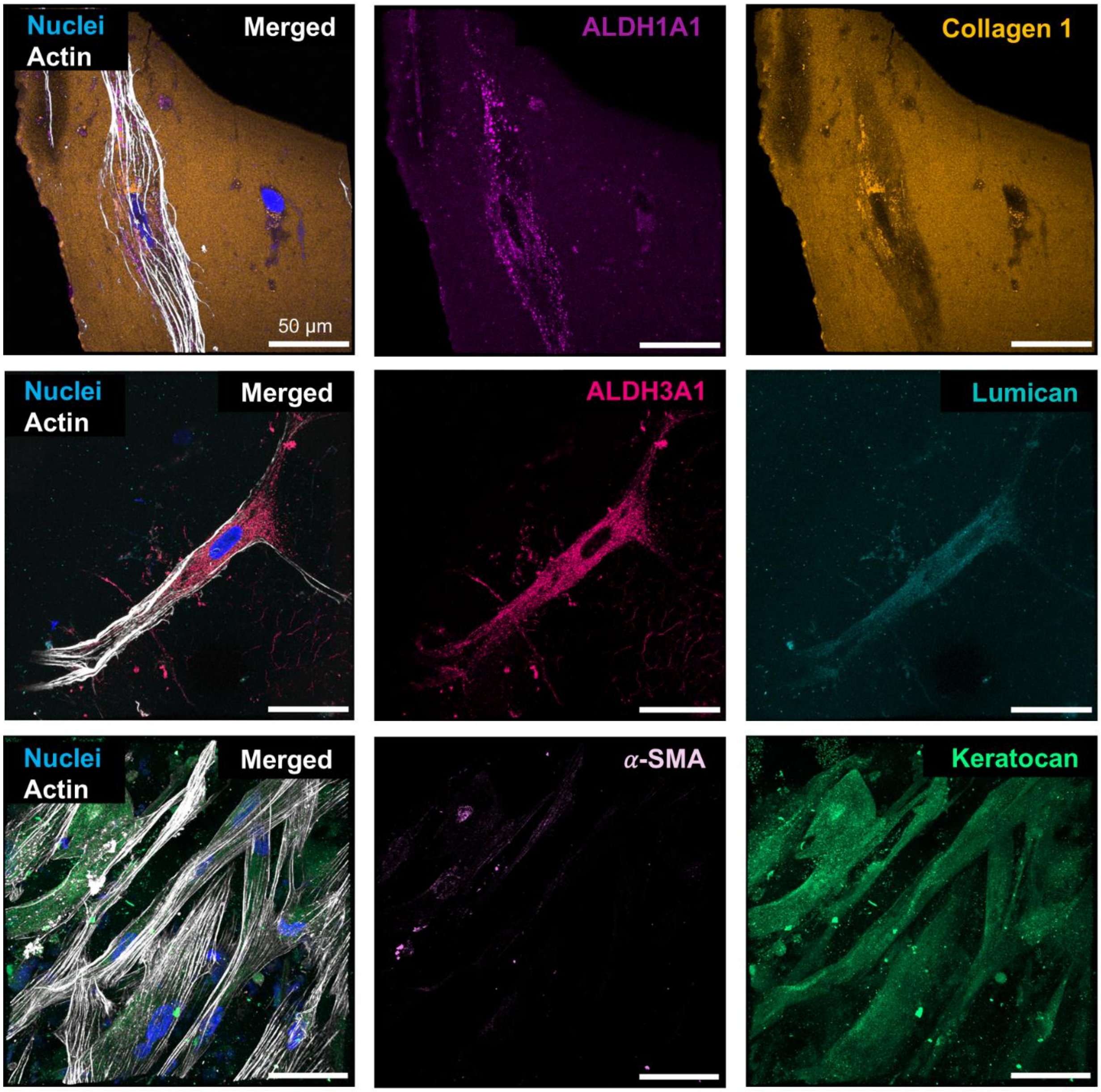

Figure S12. Immunostaining of 3D CSK-MSC encapsulated in 30 wt % RA-GelMA cultivated in Mesenpan with 2 % HS after differentiation for ALDH1A1 (light pink), Collagen 1 (orange), ALDH3A1 (dark pink), Lumican (turquoise), α-SMA (lilac), and Keratocan (green) with Hoechst Nuclear Staining (blue) and phalloidin actin staining (white). Representative images from one technical replicate per antibody couple. All conditions were performed in three technical triplicates (n = 3).
