# Supplementary material for: Toward clinically relevant automated corneal biomanufacturing with human-derived FBS alternatives
Source: Sci Rep. 2026 Jun 17;16:18874. doi: 10.1038/s41598-026-58401-5 (PMC13276165; doi:10.1038/s41598-026-58401-5)
Supplement: Supplementary file 2 — Supplementary Material 2 [file 41598_2026_58401_MOESM2_ESM.docx]

**Supplementary Information**

This Supplementary Information file contains the individual fluorescence channels corresponding to the immunofluorescence images presented in the main manuscript. Individual fluorescence channels for all immunofluorescence stainings performed in 2D cultures are provided in Supplementary Figures S1–S6. Corresponding single-channel images for 3D cultures are provided in Supplementary Figures S7–S12. These images include separate visualization of nuclear staining, cytoskeletal organization, and marker-specific signals to support the interpretation of the merged images shown in the main figures.
